# Nsun2 controls cardiac homeostasis and hypertrophic response by regulating PRKACA expression

**DOI:** 10.7150/thno.104441

**Published:** 2025-01-20

**Authors:** Dongdong Jian, Xiaolei Cheng, Datun Qi, Shixing Wang, Chenqiu Wang, Yingchao Shi, Zhen Li, Shouyi Jin, Zhen Jia, Peng Teng, Zhen Pei, Xiaoping Gu, Liguo Jian, Wengong Wang, Xia Yi, Junyue Xing, Hao Tang

**Affiliations:** 1 Department of Biochemistry and Molecular Biology, Beijing Key Laboratory of Protein Posttranslational Modifications and Cell Function, School of Basic Medical Sciences, Peking University Health Science Center, 38 Xueyuan Road, Beijing 100191, China.; 2 Department of Cardiology, Henan Province Key Laboratory for Prevention and Treatment of Coronary Heart Disease, Central China Fuwai Hospital of Zhengzhou University, Zhengzhou, Henan, 451464, China.; 3 Zhengzhou Key Laboratory of Cardiovascular Aging, Henan Key Laboratory of Chronic Disease Management, National Health Commission Key Laboratory of Cardiovascular Regenerative Medicine, Central China Fuwai Hospital of Zhengzhou University, Fuwai Central China Cardiovascular Hospital & Central China Branch of National Center for Cardiovascular Diseases, Zhengzhou, Henan, 451464, China.; 4 Department of Anesthesiology, Affiliated Drum Tower Hospital of Medical School of Nanjing University, Nanjing, Jiangsu, 210008, China.; 5 Department of Cardiology, The Second Affiliated Hospital of Zhengzhou University, Zhengzhou, Henan, 450003, China.; 6 Department of Cardiovascular Surgery, The First Affiliated Hospital, Zhejiang University School of Medicine, 79 Qingchun Road, Hangzhou 310003, China.; 7 Changzhi Medical College, Changzhi, Shanxi, 046000, China.

**Keywords:** heart hypertrophy, Nsun2, PRKACA, epitranscriptomic modification, cardiomyocyte

## Abstract

**Rationale:** Internal modifications of mammalian RNA have been suggested to be essential for the maintenance of cardiac homeostasis. However, the role of RNA cytosine methylation (m5C) in the heart remains largely unknown.

**Methods:** Bulk and single cell RNA sequencing data and tissues from the human hearts were exploited for analyzing the expression of RNA m5C modifying proteins. Neonatal rat and adult mouse cardiomyocytes were isolated to assess the impact of Nsun2 on cellular hypertrophic response. Cre/LoxP-mediated gene knockout and recombinant adeno-associated virus serotype 9 (rAAV9) were employed respectively to achieve cardiac-specific interference of the expression of related genes in mice that were subjected to heart stresses from aging, aortic constriction, and angiotensin II stimulation. RNA m5C immunoprecipitation sequencing (m5C-RIP-seq), RNA pull-down, polysome profiling, reporter gene analysis, and IonOptix measurement were conducted to elucidate the involved regulatory mechanisms.

**Results:** Nsun2 expression was significantly elevated in human, rat, and mouse hypertrophic myocardial cells. Knockout of Nsun2 (αMHC-Cre^ERT2^, Nsun2 flox^+/+^) abolished the hypertrophic response of mice to diverse stresses, while accelerating the progression of heart failure. Mechanistically, Nsun2 specifically methylates PKA catalytic subunit alpha (PRKACA) mRNA, which substantially promotes PRKACA translation in a YBX1-dependent manner. Nsun2 ablation markedly attenuated the activation of PKA signaling, as evidenced by the reduced PKA activity and protein phosphorylation levels of PKA substrates, impaired myocyte contraction and relaxation, and disturbed calcium transients. Overexpressing Nsun2 and PRKACA-3'UTR transcripts in the myocardia sensitized and desensitized heart hypertrophic responses, respectively, whereas co-administration of the PKA inhibitor H-89 or overexpressing PRKACA-3'UTR transcript lacking Nsun2 methylating regions failed to produce corresponding responses, reiterating the significance of Nsun2-PRKACA regulation in the cardiac hypertrophic program.

**Conclusion:** These observations reveal the importance of Nsun2-PRKACA regulation in cardiac homeostasis, which provides novel insights into heart function modulation and sheds light on future treatments for hypertrophic remodeling associated heart diseases.

## Introduction

In the adult heart, cardiomyocytes are terminally differentiated. Instead of proliferating, individual cardiac myocytes increase in size, and the heart develops hypertrophy to reduce ventricular wall tension and maintain cardiac output in response to an increased workload 1. Cardiac hypertrophy is induced as an adaptive response to diverse pathological stimuli, such as chronic hypertension, myocardial infarction, aortic stenosis, mitral regurgitation, storage diseases and genetic mutations in genes encoding sarcomere proteins (hypertrophic cardiomyopathy, HCM) 2. Initially, the hypertrophic reaction was considered a protective compensatory response of cardiomyocytes when coping with stress. However, hypertrophied hearts can readily progress into a decompensated state and ultimately heart failure, unless these stresses are promptly alleviated at an early stage 3. Dissecting the regulators responsible for heart hypertrophy, specifically focusing on those affecting the turning point of the chronic remodeling process, appears to be of critical importance.

Methylation is an important epitranscriptomic chemical modification that is installed at specific positions of different nucleotides of RNA, such as m6A, m5C, m5U, m1A, m6Am, m7G, etc 4. As an essential part of post-transcriptional regulation, RNA methylation regulates numerous physiological and pathological processes 5. Previous studies have reported that mRNA m6A marks are necessary for cardiac stress response modulation and homeostasis maintenance 6,7. However, other RNA methylations in the heart remain largely unexplored. Similar to m6A, RNA cytosine methylations (m5C) are in the charge of diverse 'writer' (Nsun1 - Nsun7, DNMT2), 'eraser' (TET1 and ALKBH1), and 'reader' (YBX1, ALYREF, and RAD52) proteins 8. Among these, Nsun2, originally characterized as a tRNA cytosine methyltransferase 9,10, has been extensively studied for its ability to methylate mRNA (5-methylcytosine, m5C), which in turn affects almost all aspects of RNA metabolic processes, including mRNA maturation, translation, and degradation 11,12. In this study, we observed that Nsun2 expression was significantly elevated in hypertrophied myocardium. Cardiac-specific ablation of Nsun2 abolished the stress-induced heart hypertrophic response but exacerbated heart failure progression. Through RNA m5C-sequencing profiling, the PKA signal was strikingly unveiled to be modulated by Nsun2 in the cardiomyocyte.

The PKA holoenzyme is a tetramer consisting of two regulatory (PKA-R) and two catalytic (PKA-C) subunits, among which the PKA catalytic subunit alpha (PRKACA) is the most abundant and ubiquitously expressed 13,14. Canonical PKA activation is triggered by cAMP/PKA-R binding-induced dissociation of PKA-C from the tetrameric holoenzyme. Notably, holoenzyme separation may not be necessary for PKA activation under certain circumstances. For instance, PKA-C subunits are also sequestered by the inhibitor of κB (I-κB) proteins within the I-κB-PKA-C complex, and can be activated following I-κB degradation by a plethora of stimuli, such as endothelin (ET-1), angiotensin II (AngII), lipopolysaccharides (LPS), and interleukin-1 (IL-1) 15,16. Hence, the activation of PKA signaling depends primarily on the levels of free PKA-C subunits. Nevertheless, current knowledge of the regulation of PKA-C subunit gene expression, which directly determines the levels of free PKA-C subunits, is limited. PKA signaling plays a fundamental role in regulating cardiac performance and morphology. Under hypertrophic stresses, PKA phosphorylates target proteins in discrete microdomains to increase cardiac size, inotropy (contraction), and lusitropy (relaxation). For example, PKA phosphorylates cAMP-response element binding protein (CREB) at Ser133 in the nuclei of cardiomyocytes, thus activating CREB-mediated transcription of hypertrophy-related genes 17-19. In addition, phosphorylation of phospholamban (PLN) at Ser16 increases the activity of the sarcoplasmic reticulum (SR) Ca^2+^ ATPase (SERCA) to increase SR Ca^2+^ uptake 20,21, while phosphorylation of ryanodine receptor type 2 (RyR2) enhances SR Ca^2+^ release 22-24. Phosphorylation of these calcium cycling proteins apparently intensifies Ca^2+^ transients as well as cardiac contractility. Moreover, phosphorylation of cTnI at Ser23/24 by PKA weakens the cTnI-cTnC interaction, thereby promoting dissociation of Ca^2+^ from cTnC and accelerating myofibril relaxation 25-27. These crucial regulatory events suggest that PKA activation is required for the cardiac adaptive response to hypertrophic stimuli, which is also evidenced by the preceding report that depressed PKA activity impairs isoproterenol-induced heart and cardiomyocyte contractility 28.

Mechanistically, our investigation revealed that Nsun2 facilitates PRKACA translation through mRNA methylation, which enhances cardiac PKA signaling and subsequent adaptive responses, including increases in myocyte size, inotropy, and lusitropy, under conditions of hypertrophic stress. Inhibition of Nsun2-PKA signaling, either directly through the PKA inhibitor H-89 or by overexpressing PRKACA-3'UTR transcript that competitively inhibits the methylation of PRKACA mRNA by Nsun2, attenuated corresponding cardiac hypertrophic responses. These findings contribute to a more comprehensive understanding of the role of RNA epigenetic modifications in cardiac homeostasis and pathogenesis.

## Results

### Nsun2 expression is induced in the hypertrophied myocardium

**Figure 1:**
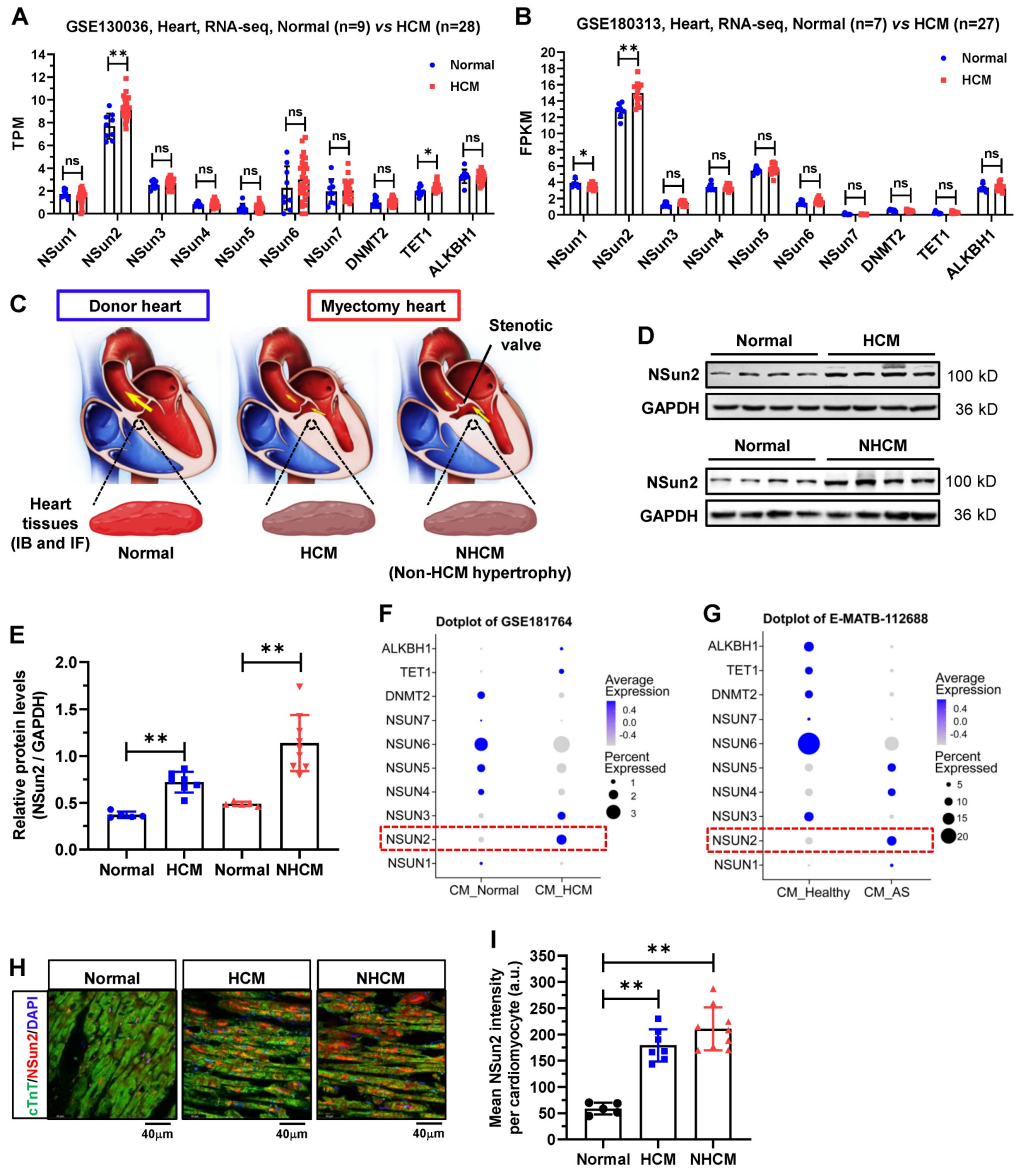
** Nsun2 expression is increased in human hypertrophied myocardium. (A and B)** Expressions of RNA m5C 'writer' and 'eraser' proteins were examined in two RNA-sequencing datasets including donors with normal myocardium (GSE130036, Normal, n = 9; GSE180313, Normal, n = 7) and patients with hypertrophic cardiomyopathy (GSE130036, HCM, n = 28; GSE180313, HCM, n = 27). **(C)** Schematic illustration of the specimen sources (Normal, healthy donors; HCM, patients with hypertrophic cardiomyopathy; NHCM, patients with aortic stenosis alongside left ventricular hypertrophy) (upper panel). **(D)** Immunoblotting analysis of Nsun2 expression in ventricle tissues described in (C). **(E)** Densities of the blotting signals in (D) were scanned and plotted. Normal, n = 5; HCM, n = 7; NHCM, n = 9. **(F and G)** Expressions of RNA m5C 'writer' and 'eraser' proteins were examined in cardiomyocytes in two single cell RNA sequencing (scRNA-seq) datasets (GSE181764: single nucleus RNA-sequencing (snRNA-seq) of 2 unused donor hearts (CM_Normal), 1 obstructive HCM specimen, and 6 non-obstructive HCM specimens (CM_HCM); E-MATB-112688: scRNA-seq of 5 patients with severe aortic valve stenosis (CM_AS) and 14 healthy hearts (CM_Healthy)). **(H)** Representative images of immunofluorescence staining of corresponding heart sections (lower panel). Nuclei, DAPI (blue); cardiomyocytes, cTnT (green); Nsun2 (red). Scale bar, 40 µm. **(I)** The mean fluorescence intensity of Nsun2 per cardiomyocyte in (H) was quantified and plotted. Three microscopic fields were included for statistics of mean intensity every tissue sample. Normal, n = 5; HCM, n = 7; NHCM, n = 9. Data were represented as means ± SEMs and analyzed by unpaired Student's t-test. **P < 0.01.

To unveil the potential role of RNA m5C modifications in the heart, we preliminarily examined expressions of thus far identified m5C modifier proteins in the human hypertrophied myocardium by reanalyzing the RNA sequencing data deposited in datasets GSE130036 and GSE180313, both of which comprise normal and HCM left ventricle samples 29,30. As illustrated in Figure 1A-B, with the exception of Nsun2, the majority of the modifiers exhibit no changes in their expressions between normal and HCM ventricle tissues. Correspondingly, Nsun2 expression was observed to be significantly up-regulated in HCM patients compared with healthy individuals. To substantiate the expression changes of Nsun2 in myocardial hypertrophy, human heart tissues procured from healthy donors (normal) and patients with HCM or non-HCM hypertrophy (NHCM) were collected (Figure 1C and Table 1). Consistent with the sequencing results, an elevated Nsun2 protein level was observed in the myocardium from HCM and NHCM patients compared to that from the healthy donors (Figure 1D-E). Furthermore, two snRNA-seq datasets of human hypertrophied hearts, corresponding to HCM (GSE181764) and NHCM (E-MATB-112688) hypertrophy respectively, were utilized to examine whether the altered expression of Nsun2 occurs in the myocardial cell 31,32. Indeed, Nsun2 expression was found to increase significantly in HCM (Figure 1F) and NHCM (Figure 1G) cardiomyocytes when compared to healthy ones. Through immunostaining analysis of the obtained heart tissues, the elevation of Nsun2 expression in the cardiac myocytes undergoing hypertrophy was confirmed (Figure 1H-I).

**Figure 2:**
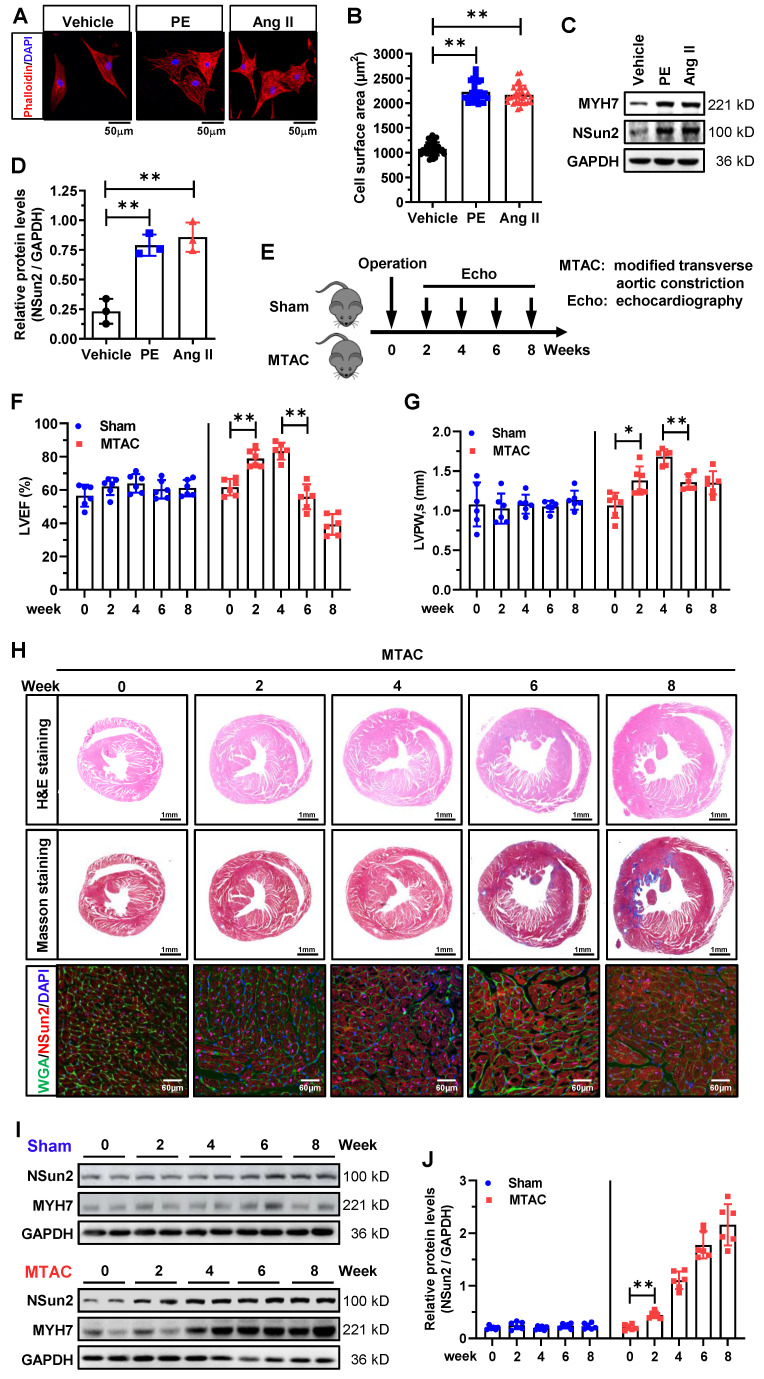
** Nsun2 expression is provoked in stress-induced cardiomyocyte and myocardial hypertrophy. (A to D)** Neonatal rat ventricular myocytes (NRVMs) were treated with PE (100 µM) or Ang II (1 µM) for 48 hours to induce cellular hypertrophy. Representative images of cell morphology stained by phalloidin (A); quantification of the average cell surface area of NRVMs in response to Vehicle, PE or Ang II treatment, n ≥ 30 cells per group (B); western blotting analysis of the expressions of Nsun2, MYH7 and GAPDH (C); statistical analysis of the scanned densities by unpaired Student's t-test and plotting as means ± SDs from 3 independent experiments (D). **(E)** Schedule of the mouse model of heart hypertrophy induced by a modified transverse aortic constriction (MTAC, constriction with the diameter of 0.51 mm). **(F and G)** Summarized echocardiographic measurements of left ventricular EF (F), and LVPW,s (G) from Sham and MTAC groups at the indicated weeks. n = 6 for each group.** (H)** Representative images of the histochemistry (H&E, upper panel; Masson, middle panel) and immunofluorescence (WGA, green; Nsun2, red; DAPI, blue; lower panel) staining of murine heart sections. **(I)** Immunoblotting analysis of the expressions of Nsun2, MYH7 and GAPDH from Sham and MTAC groups at the indicated weeks. **(J)** Densities of the blotting signals in (I) were scanned and plotted. n = 6 for each group. Data were analyzed by one-way ANOVA followed by Bonferroni's post hoc tests and represented as means ± SEMs. *P < 0.05, **P < 0.01.

Subsequently, we investigated Nsun2 expression in neonatal rat ventricular myocytes (NRVMs) exposed to phenylephrine (PE) or angiotensin II (AngII) treatment. Phalloidin staining demonstrated that cardiomyocyte hypertrophy was effectively induced by PE or AngII (Figure 2A-B). Concurrent with the increase in hypertrophy marker protein levels of MYH7, Nsun2 expression was drastically induced in these cultured hypertrophic myocytes (Figure 2C-D). To the best of our knowledge, the stress-driven remodeling of cardiac hypertrophy into heart failure is a chronic and staged process. To accurately elucidate the potential role of Nsun2 in the cardiac remodeling course, we developed a modified transverse aortic constriction (MTAC)-induced cardiac hypertrophy mouse model with a reduced aortic constriction degree (MTAC, 25-gauge needle, outer diameter 0.51mm; traditional TAC, 27-gauge needle, outer diameter 0.41mm; Figure S1A-B), which would better recapitulate the remodeling stages 33,34. As indicated in Figure 2E, the hypertrophic progression was scrutinized in consecutive weeks after the surgical operation. Through echocardiographic evaluations, the remodeling phases for heart compensation and decompensation in response to the pressure overload were easily determined, showing that the MTAC heart progressed into a decompensated state at around 6 weeks post-operation (Figure 2F-G, Figure S1C-D). In addition, the heart weight/tibia length ratio (HW/TL) in MTAC mice was observed not to increase further at 6 weeks, reconfirming this turning point of heart adaptations to MTAC stresses (Figure S1E). These morphological and functional changes were also visibly identified by haematoxylin-eosin (H&E) and Masson-trichrome staining of murine heart ventricles (Figure 2H, upper and middle panels). Consistent with the observations in human specimens, Nsun2 expression was signally induced in MTAC hypertrophic myocytes (Figure 2H, lower panel) and myocardia (Figure 2I-J), even earlier than the hypertrophy indicator protein MYH7 at 2 weeks post-operation. Jointly, these results suggest that the expression of RNA cytosine methyltransferase Nsun2 is stimulated in the hypertrophied heart, indicating its probable involvement in the regulation of cardiac hypertrophic remodeling.

### Postnatal knockout of murine cardiac Nsun2 eliminates aging-induced myocardial cell hypertrophy and impairs heart function in aged mice

**Figure 3:**
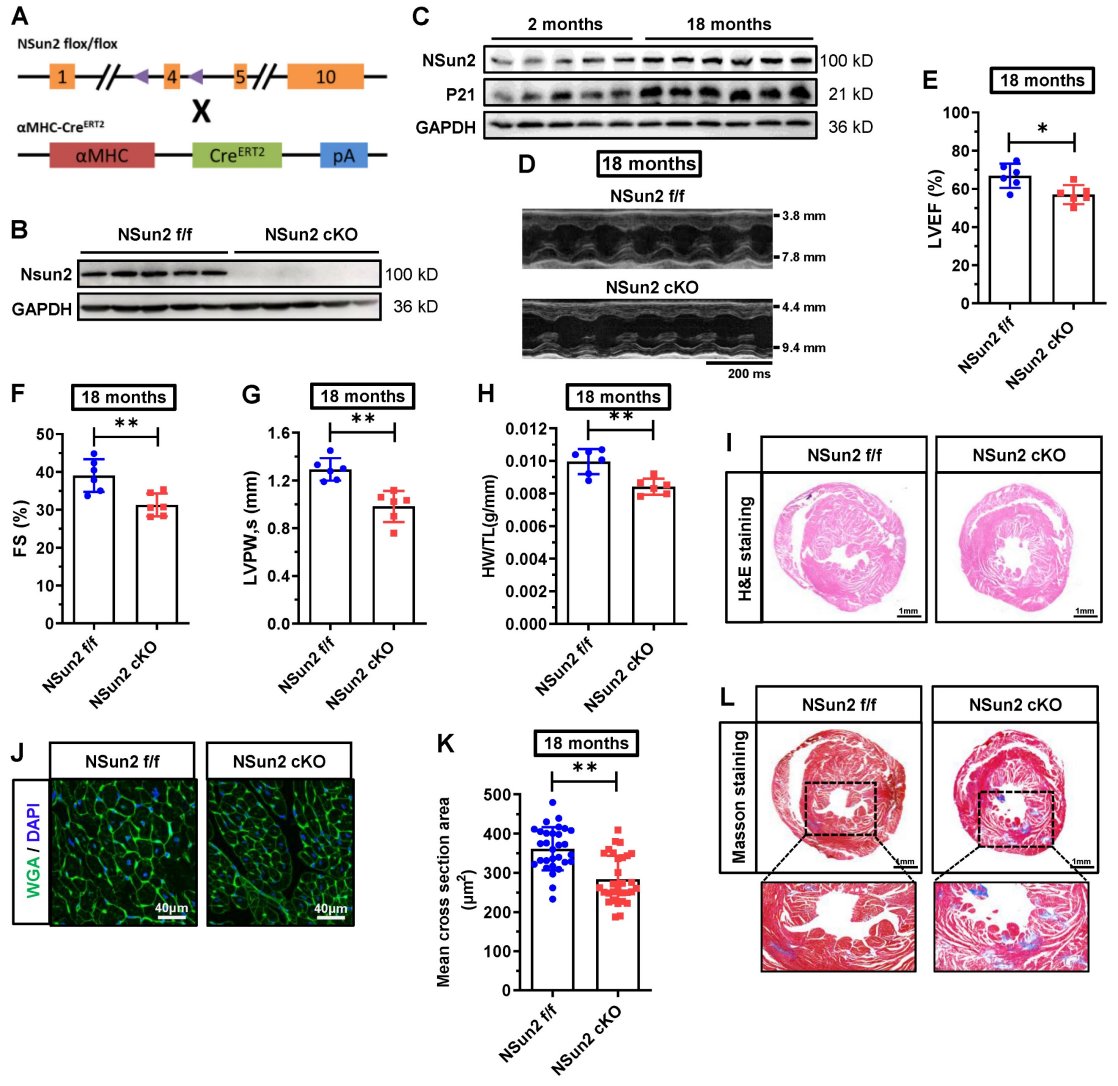
** Cardiomyocyte-specific knockout of Nsun2 eliminates aging-induced myocardial cell hypertrophy and impairs heart function in aged mice. (A)** Overview of the targeting strategy to create cardiomyocyte-specific Nsun2 knockout mice by CRISPR/Cas-mediated genome engineering and mating with αMHC-Cre^ERT2^ mice. **(B)** Immunoblotting assessment of the knockout efficiency of Nsun2 using Langendorff-isolated cardiomyocytes. **(C)** Immunoblotting analysis of the expressions of Nsun2 and P21 in young (2 months) and aged mice (18 months). **(D)** Representative echocardiographic images of control (Nsun2 f/f) and cardiomyocyte-specific Nsun2 knockout (Nsun2 cKO) mice at 18 months of their age. **(E to G)** Summarized echocardiographic measurements of left ventricular EF (E), FS (F), and LVPW,s (G) of the mice described in (D), n = 6 for each group. **(H)** Statistical results of murine HW/TL values, n = 6 for both groups. (I) Representative images for H&E staining of ventricles. Scale bar, 1 mm. **(J and K)** Representative pictures of WGA staining of murine heart sections. Scale bar, 40μm. (J) and quantification of the mean cross-sectional areas of the stained cardiomyocytes (K); n ≥ 30 cells per group. **(L)** Representative images for Masson staining of ventricles. Scale bar, 1 mm. Data were represented as means ± SEMs and analyzed by unpaired Student's t-test. *P < 0.05, **P < 0.01.

To decipher the function of Nsun2 in the heart, Nsun2 was specifically knocked out in the myocardium in 2-month-old mice, as depicted in Figure 3A. Immunoblotting analysis of Langendorff-isolated cardiomyocytes suggested that Nsun2 was almost entirely absent in the myocardium in Nsun2 knockout (Nsun2 cKO: αMHC-Cre^ERT2^, Nsun2 flox^+/+^) mice (Figure 3B). Potential cardiac phenotype alterations in Nsun2 knockout mice were monitored using monthly echocardiography. At 8 months of age, a comprehensive evaluation of the heart, including echocardiography, HW/TL measurement, and H&E and wheat germ agglutinin (WGA) staining, was conducted. However, no significant differences in heart morphology, weight, function, or cardiomyocyte size were observed between Nsun2 cardiac-deficient mice and their littermate controls (Nsun2 f/f: Nsun2 flox^+/+^) (Figure S2A-J). Subsequently, these mice were assessed until their aging stages of 18 months old. With aging, the murine cardiac myocyte underwent hypertrophic growth (Figure S3A-B). Concomitantly, Nsun2 expression was observed to increase as anticipated in the aged heart (Figure 3C). In contrast to the 8-month-old mice, cardiac knockout of Nsun2 (Figure 3D) significantly reduced the 18-month mouse ejection fraction (LVEF, Figure 3E), fraction shortening (FS, Figure 3F), the thickness of left ventricular anterior (LVAW) and posterior (LVPW) walls (Figure 3G and Supplementary Figure 3C), heart weight (Figure 3H), and the size of both heart (Figure 3I) and myocardial cells (Figure 3J-K), and increased their internal dimension of the left ventricle (LVID, Figure S3D), cardiac aging progression (indicated by the expression changes of P53 and P21, Figure S3E), and myocardial interstitial fibrosis (Figure 3L). These data suggested that cardiac deficiency of Nsun2 impaired the old mouse heart function and hypertrophic adaptations to the aging stress.

### Nsun2 ablation in the myocardium interrupts the heart hypertrophic response and predisposes the heart to failure

**Figure 4:**
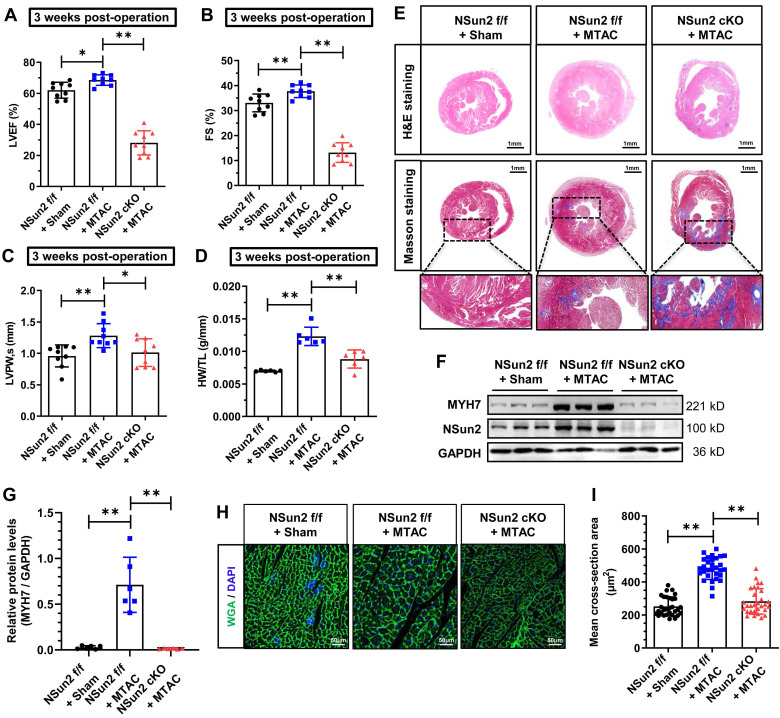
** Nsun2 ablation in the myocardium interrupts the heart hypertrophic response and predisposes the heart to failure.** Mice were divided into three groups including 'Nsun2 f/f + Sham', 'Nsun2 f/f + MTAC' and 'Nsun2 cKO + MTAC'. The following analysis was performed at 3 weeks after surgery. **(A to C)** Summarized echocardiographic measurements of left ventricular EF (A), FS (B), LVPW,s (C) of the mice as described above, n = 9 for each group. **(D)** Statistical results of murine HW/TL values. n = 6 for each group. **(E)** Representative images of histological analysis of ventricles by H&E and Masson staining. Scale bar, 1 mm, and 400 µm for the magnification of Masson staining. **(F and G)** Immunoblotting analysis of the expression changes of Nsun2, MYH7 and GAPDH in murine heart tissues (F) and densities of the blotting signals for MYH7 were scanned and plotted (G). **(H)** WGA staining of ventricle sections from the three indicated groups. WGA, green; DAPI, blue; Scale bar, 50µm. **(I)** Statistical analysis of the mean cross-sectional areas of the stained cardiomyocytes from (H); n ≥ 30 cells per group. Data were analyzed by one-way ANOVA followed by Bonferroni's post hoc tests and represented as means ± SEMs. *P < 0.05, **P < 0.01.

A growing body of evidence indicates that certain regulators in the heart are specifically deployed to address particular stresses and are necessary for maintaining heart homeostasis 35,36. Inspired by this, we next investigated the effects of Nsun2 knockout in the heart when subjected to pathological hypertrophic pressures. Echocardiographic images revealed impaired ventricular motion in Nsun2 cKO mice as early as 3 weeks after MTAC, whereas this defect was not observed in Nsun2 f/f mice receiving MTAC operation (Figure S4A), the hearts of which were considered to be experiencing compensation as previously mentioned. Data obtained from echocardiography demonstrated that murine heart hypertrophic remodeling was successfully induced by the increased afterload (MTAC), as manifested by the compensatory potentiation of cardiac function (Figure 4A-B), left ventricular wall thickening (Figure 4C and Figure S4B), and a reduction in the left ventricular chamber diameter (Figure S4C-D). However, these remodeling alterations were abruptly halted by Nsun2 knockout, which exacerbated pressure overload-induced heart failure development (Figure 4A-C, Figure S4A-E). Additionally, Nsun2 deficiency was observed to remarkably repress the increase in HW/TL in MTAC mice (Figure 4D). To corroborate these morphological and structural changes, H&E staining and Masson-trichrome staining were performed on the murine hearts, and the results indicated that MTAC minimally triggered the heart hypertrophic response in the absence of Nsun2 (Figure 4E, upper panel, H&E staining) but aggravated interstitial fibrosis of the Nsun2-devoid myocardium (Figure 4E, middle and lower panels, Masson staining), confirming the acceleration of heart failure progression. Immunoblotting analysis of Langendorff-isolated cardiomyocytes indicated that Nsun2 ablation markedly diminished MYH7 expression in MTAC mouse cardiomyocytes (Figure 4F-G), suggesting a defect in response to the hypertrophy stress. Consistent with the immunoblotting results, the increases in cardiomyocyte dimensions in MTAC mice were significantly attenuated by knocking out Nsun2 (Figure 4H-I).

**Figure 5:**
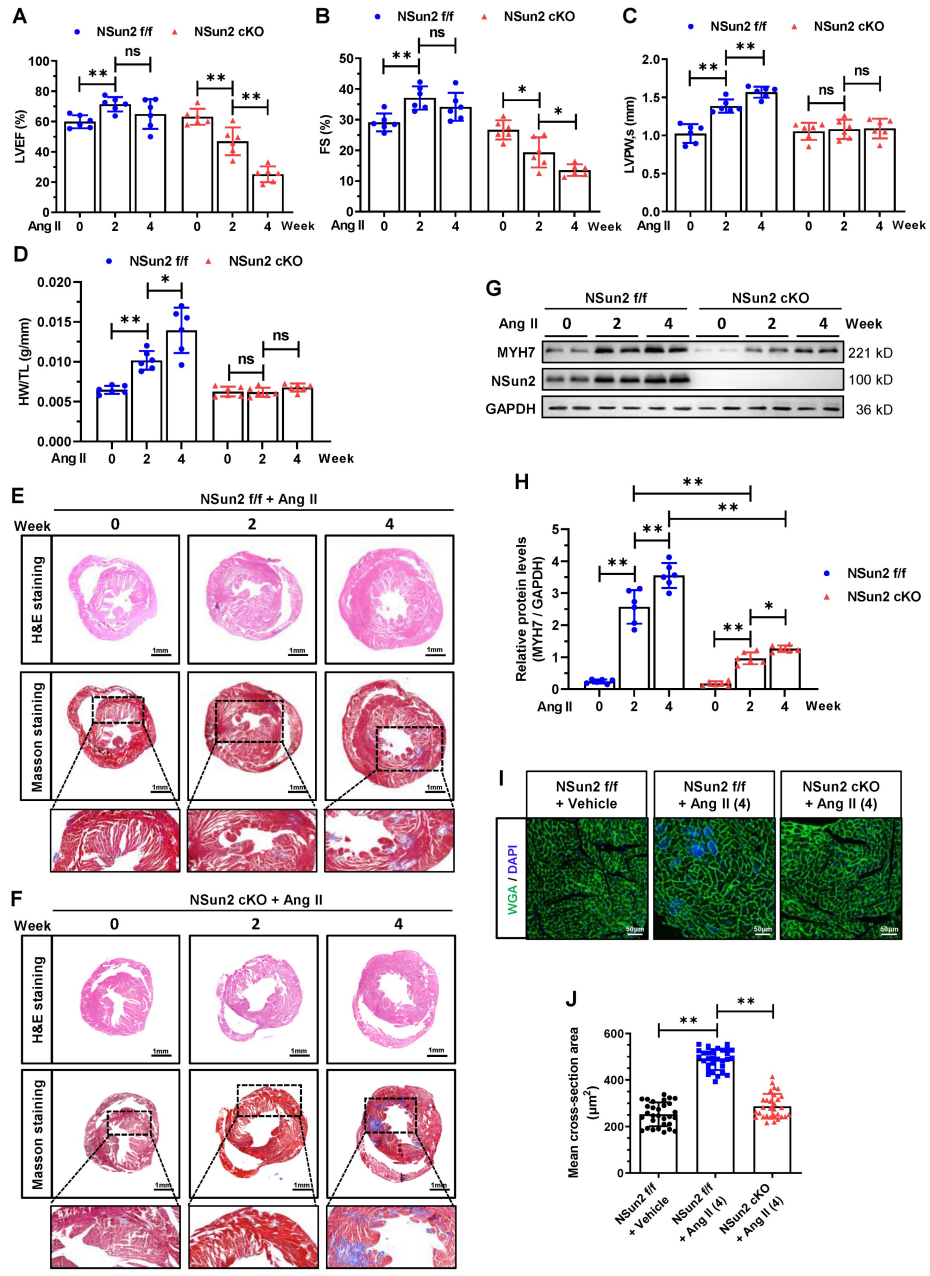
** Nsun2 deficiency in the myocardium interrupts AngII-induced heart hypertrophic response.** Nsun2 f/f and Nsun2 cKO mice were administrated by subcutaneous infusion of AngII at a dose of 1000 ng/kg/min for 0, 2 and 4 weeks. **(A to C)** Summarized echocardiographic measurements of left ventricular EF (A), FS (B), LVPW,s (C) of the mice at the indicated time points, n = 6 for each group. (D) Statistical results of murine HW/TL values. n = 6 for each group. **(E and F)** Representative images of histological analysis of ventricles by H&E and Masson staining. Scale bar, 1 mm, and 400 µm for the magnification of Masson staining. **(G and H)** Immunoblotting analysis of the expression changes of Nsun2, MYH7 and GAPDH in the indicated murine heart tissues (G) and densities of the blotting signals for MYH7 were scanned and plotted (H). **(I)** WGA staining of ventricle sections from Nsun2 f/f and Nsun2 cKO mice administrated by subcutaneous infusion of AngII for 4 weeks. WGA, green; DAPI, blue; Scale bar, 50µm. **(J)** Statistical analysis of the mean cross-sectional areas of the stained cardiomyocytes from (I); n ≥ 30 cells per group. Data were analyzed by one-way ANOVA followed by Bonferroni's post hoc tests and represented as means ± SEMs. ns, no significance. *P < 0.05, **P < 0.01.

Moreover, we established an angiotensin II (AngII)-induced heart hypertrophy mouse model to further substantiate the role of cardiac Nsun2 in response to hypertrophic stresses. With the induction of AngII, the progression of heart hypertrophic remodeling was documented in Nsun2 f/f mice, as evidenced by the time-dependent changes in LVEF, FS, LVAW, LVPW, LVID, and HW/TL values (Figure 5A-D, Figure S4F-H). Upon deletion of Nsun2 (Nsun2 cKO) in the myocardium, these progressive alterations were nearly abolished or reversed (Figure 5A-D, Figure S4F-H) except serum NT-proBNP levels that were significantly elevated (Figure S4I), demonstrating suspended cardiac hypertrophy and acceleration of heart failure development.

Consistent with these observations, the ventricle size and wall thickening degree were significantly reduced in AngII-treated Nsun2 cKO mice, while the interstitial fibrosis of their myocardia was greatly increased when compared to these indices in Nsun2 f/f mice that received AngII treatment (Figure 5E-F). At the molecular and cellular level, Nsun2 knockout attenuated myocardial cell induction of hypertrophy indicator MYH7 to a large extent (Figure 5G-H), and significantly reduced its enlargement and hypertrophy (Figure 5I-J) in the AngII-induced hypertrophied heart. Based on these observations, we conclude that deprivation of cardiac Nsun2 abrogates the heart hypertrophic response to pathological stresses and predisposes the heart to failure.

### Nsun2 methylates the mRNA of PKA catalytic subunit alpha (PRKACA)

**Figure 6:**
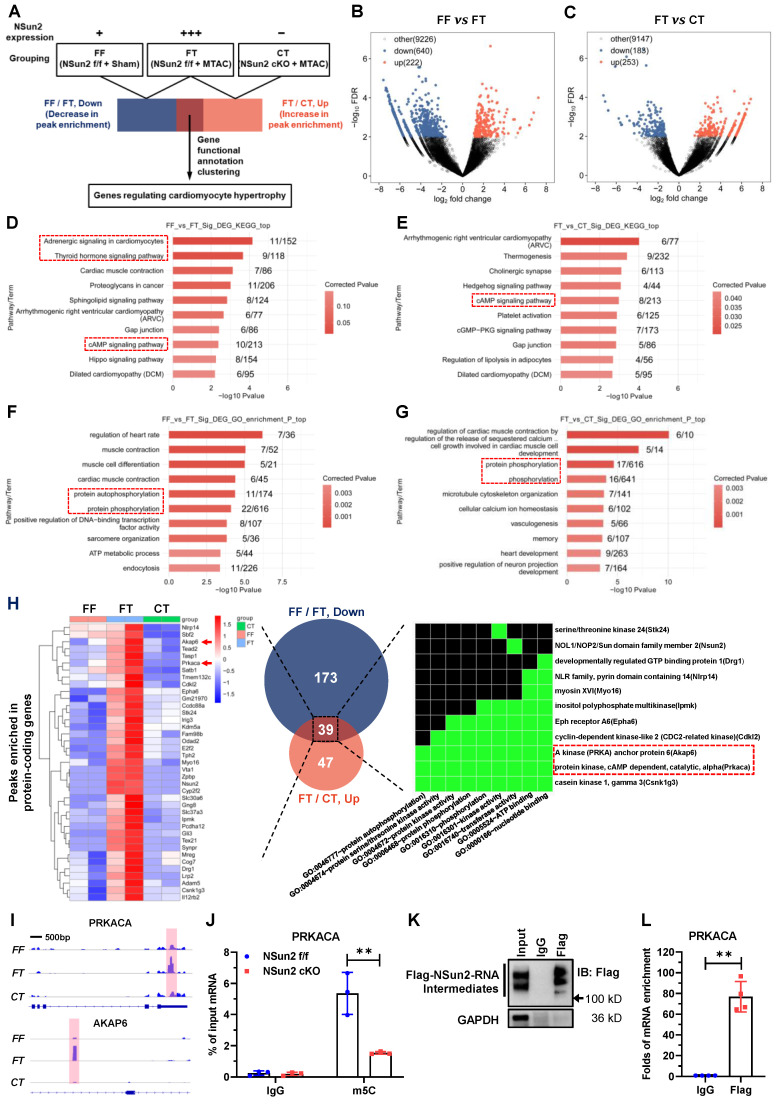
**Nsun2 methylates the mRNA of PKA catalytic subunit alpha (PRKACA). (A)** Screening strategy for genes responsible for the regulation of Nsun2 on cardiac hypertrophy. **(B and C)** Volcano plotting of the differentially enriched m5C peaks between FF (Nsun2 f/f + Sham) and FT (Nsun2 f/f + MTAC) groups (B), and between FT and CT (Nsun2 cKO + MTAC) groups (C). **(D to G)** Genes with differentially enriched m5C peaks described in (B) and (C) were analyzed by KEGG signaling pathway (D and E) and GO functional annotation (F and G), respectively. **(H)** Cross-referencing the protein-coding genes from the datasets of 'FF/FT, Down' and 'FT/CT, Up' obtained in (B) and (C) (middle), then heat map illustration (left) and gene functional annotation clustering (right) of the filtered common genes. **(I)** IGV view of the m5C peaks annotated to the gene body of PRKACA and AKAP6, respectively. **(J)** m5C-RIP-qPCR was performed on isolated cardiomyocytes from Nsun2 f/f and Nsun2 cKO mice using an m5C antibody. **(K)** Immunoprecipitation assessment of Flag-Nsun2-RNA intermediates (Nsun2-MeRIP assay) formed in murine HL-1 cardiac cells receiving the transfection of constructs expressing mutant Nsun2. **(L)** The enriched PRKACA mRNA in Nsun2-MeRIP materials was quantified by real-time qPCR. Data were analyzed by unpaired Student's t-test and represented as means ± SDs from ≥ 3 independent experiments. **P < 0.01.

To unveil the underlying mechanisms by which Nsun2 regulates the cardiac hypertrophic response, RNA m5C-RIP-seq (RNA m5C immunoprecipitation followed by sequencing) was carried out using Langendorff-obtained cardiomyocytes from the FF (Nsun2 f/f + Sham), FT (Nsun2 f/f + MTAC) and CT (Nsun2 cKO + MTAC) groups. Aiming to identify potential Nsun2 substrate genes, the following sequencing analysis was conducted mainly between groups with differential expression of Nsun2, including the FF and FT groups and the FT and CT groups (Figure 6A).

Volcano plotting showed that MTAC resulted in 862 differentially enriched m5C peaks (Figure 6B; up, increase in enrichment, 222 peaks; down, decrease in enrichment, 640 peaks), and Nsun2 knockout yielded 436 differentially enriched m5C peaks (Figure 6C; up, increase in enrichment, 253 peaks; down, decrease in enrichment, 188 peaks). KEGG pathway analysis of the genes with differentially enriched peaks demonstrated that RNA m5C modification modulates a myriad of essential cardiac signals, among which the cAMP signaling pathway was notable (Figure 6D-E) due to its well-recognized role in cardiac hypertrophic remodeling 37. PKA-mediated phosphorylation regulation is known to be the main effector downstream of cAMP signaling in cardiomyocytes 38. Corresponding to this, GO analysis also revealed protein phosphorylation as a major biological process involved in the modulation of RNA cytosine methylation (Figure 6F-G).

To identify the core mediator in charge of heart hypertrophic regulation by Nsun2, we primarily filtered protein-coding genes for which the m5C peak enrichment degree was positively correlated with Nsun2 expression. In this way, two subsets of protein-coding genes (Table S1 and S2, FF/FT, down *vs.* FT/CT, up) were obtained from the 'FF *vs.* FT' and 'FT *vs.* CT' datasets described in Figure 6B-C, respectively. We further cross-referenced the two gene lists, and 39 common genes most likely accounting for the regulatory effect of Nsun2 on cardiac hypertrophy were ultimately acquired. Functional annotation clustering of these genes using the DAVID database 39 indicated that GO terms associated with kinases and protein phosphorylation were specifically enriched, and two constituent members of the cAMP signaling pathway, PKA catalytic subunit alpha (PRKACA) and A kinase anchor protein 6 (AKAP6), were conspicuously noted (Figure 6H). Subsequently, the differentially enriched m5C peaks of PRKACA and AKAP6 were visualized with IGV software. As shown in Figure 6I, Nsun2 knockout significantly decreased MTAC-induced enrichment of m5C peaks on either the PRKACA or AKAP6 gene. Notably, induced m5C peaks were in different regions of corresponding transcripts, with those of PRKACA in the 3' untranslated region (3'UTR) and those of AKAP6 in the intronic region. Given the dominant role of PRKACA in cAMP-PKA signaling cascades, we next mainly focused on PRKACA modulations.

RNA m5C immunoprecipitation followed by quantitative PCR (m5C-RIP-qPCR) was exploited to verify the methylation deposited on PRKACA mRNA and suggested that depletion of Nsun2 markedly diminished cytosine methylation levels of PRKACA mRNA in murine cardiomyocytes (Figure 6J). To substantiate the direct methylation of PRKACA mRNA by Nsun2, we mutated the conserved cysteine responsible for releasing RNA substrates in Nsun2 methyltransferase 40,41 and then performed Nsun2-mediated methylation-specific RNA immunoprecipitation (Nsun2-MeRIP) in HL-1 cardiomyocytes expressing the abovementioned Nsun2 mutant. Formation of masses of Nsun2-RNA intermediates suggested that mutated murine Nsun2 successfully trapped its RNA substrates (Figure 6K). Afterwards, qPCR assessment specifically demonstrated that PRKACA mRNA was enormously precipitated (~ 77-fold to IgG) by the Nsun2 mutant (Figure 6L), validating the direct methylation of PRKACA mRNA by Nsun2. Altogether, these results indicate that RNA m5C modification may play an important role in regulating cardiac function and homeostasis and that Nsun2-mediated methylation of PRKACA mRNA is most likely involved in heart hypertrophic remodeling.

### Nsun2 promotes PRKACA translation in a YBX1-dependent manner

**Figure 7:**
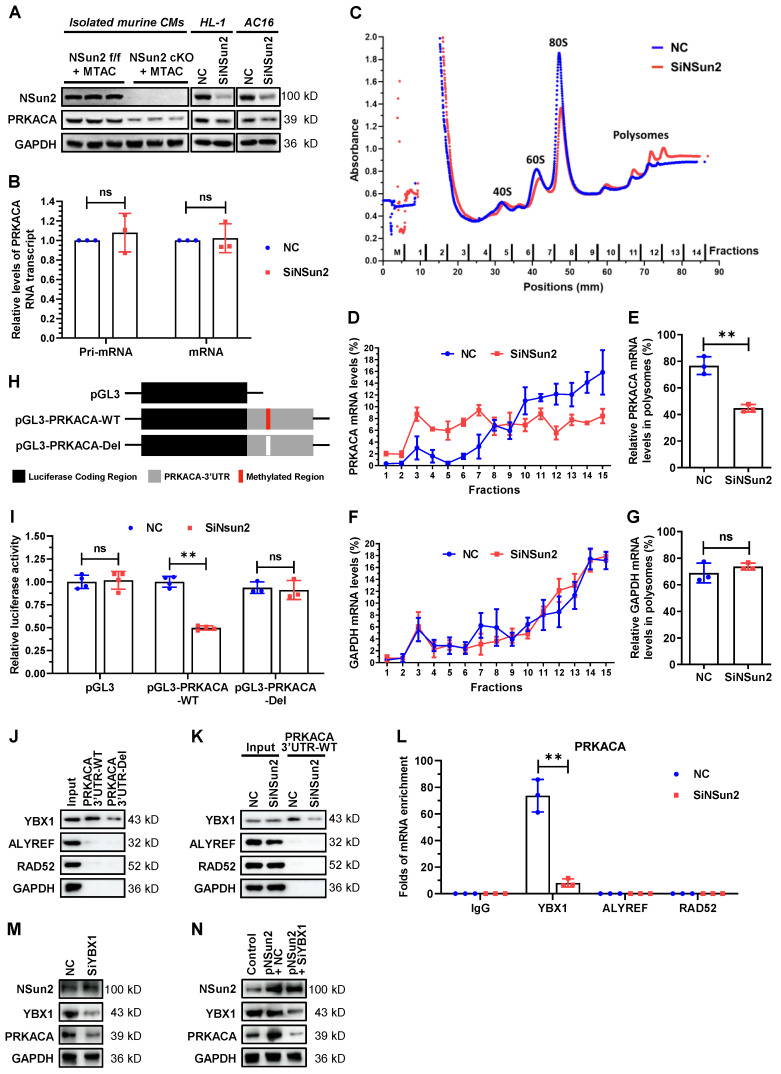
** Nsun2-mediated mRNA methylation promotes PRKACA translation in a YBX1-dependent way. (A)** Immunoblotting analysis of the expression changes of Nsun2, PRKACA and GAPDH in primary cardiomyocytes isolated from 'Nsun2 f/f + MTAC' and 'Nsun2 cKO + MTAC' mice, as well as in HL-1 and AC16 cardiac cells receiving negative control (NC) or Nsun2 (SiNsun2) siRNA. **(B)** Real-time qPCR analysis of the relative levels of PRKACA primary (Pri-) and mature mRNA transcripts in HL-1 cells between NC and SiNsun2 groups. **(C)** Polysome profiling of the HL-1 cells described in (B). **(D to G)** RNAs in the fractions obtained from (C) were extracted and subjected to qPCR analysis to quantify the relative levels of PRKACA and GAPDH mRNAs (D and F), and both of their percentages in the polysome fractions (fraction 10-14) were calculated (E and G). **(H)** Schematic diagram of luciferase reporter constructs containing the 3' untranslated region (3'UTR) of PRKACA mRNA with (pGL3-PRKACA-WT) or without (pGL3-PRKACA-Del) Nsun2 methylation region. **(I)** Dual luciferase activity analysis of the generated reporter constructs within HL-1 cells receiving Nsun2 siRNA or not. **(J)** RNA pull-down analysis with HL-1 cell lysates and PRKACA 3'UTR transcript bearing Nsun2 methylation regions or not (PRKACA 3'UTR-WT and PRKACA 3'UTR-Del). **(K)** RNA pull-down analysis using PRKACA 3'UTR transcript and lysates prepared from HL-1 cells receiving negative control (NC) or Nsun2 (SiNsun2) siRNA. **(L)** RNA immunoprecipitation within HL-1 cells in the presence of Nsun2 or not (NC and SiNsun2) using IgG, YBX1, ALYREF, and RAD52 antibodies, respectively. **(M and N)** Immunoblotting assessment of the expression changes of Nsun2, YBX1, PRKACA, and GAPDH in HL-1 cells when knocking down YBX1 alone (M), or overexpressing Nsun2 accompanied by silencing YBX1 or not (N). Data were analyzed by unpaired Student's *t*-test and shown as the means ± SDs from ≥ 3 independent experiments. ns, no significance; **P < 0.01.

We next sought to elucidate the regulation of Nsun2 on PRKACA. Immunoblotting showed that depletion of Nsun2 significantly reduced PRKACA protein expression in primary (Langendorff-isolated) or immortalized (HL-1) murine cardiomyocytes as well as in human AC16 cardiomyocytes (Figure 7A), hinting a conservation in this regulation among species. To dissect the regulatory level, we further analyzed the expression changes of PRKACA primary and mature mRNA upon Nsun2 knockdown within HL-1 cells and found that both were unaltered (Figure 7B). Then, we tested whether Nsun2 influences the translational process of PRKACA mRNA. Polyribosome profiling was conducted with materials prepared from HL-1 cells transfected with control (NC) and Nsun2 (SiNsun2) siRNA (Figure 7C). Quantitative analysis suggested that PRKACA mRNA was notably less occupied in the polysomes after knockdown of Nsun2, whereas GAPDH mRNA was unaffected (Figure 7D-G), indicative of impaired translation of PRKACA mRNA in Nsun2-deficient HL-1 cells. To determine whether Nsun2 promotes PRKACA expression via RNA methylation, we generated reporter genes bearing PRKACA RNA methylation regions or not, as schematically described in Figure 7H. Afterwards, these reporter constructs were separately transfected into HL-1 cells treated with control or Nsun2 siRNA. The measured data showed that Nsun2 knockdown overtly decreased the luciferase activity in cells transfected with the pGL3-PRKACA-WT construct, but had no influence on that in cells transfected with the pGL3 or pGL3-PRKACA-Del construct (Figure 7I), attesting to the dependence on RNA methylation for PRKACA regulation by Nsun2. To identify the modified sites harbored within the 3'UTR of PRKACA mRNA, we conducted the RNA bisulfite-PCR (BS-PCR) assay using RNA isolated from HL-1 myocardial cells treated with or without Nsun2 siRNA. Through the amplification of partially overlapping RNA segments designed to comprehensively cover the methylation region, we identified the potential cytosine site specifically methylated by Nsun2 (Figure S5A-B). Notably, at least three cytosine sites (C1455, C1467, and C1587) appear to be methylated by Nsun2 in this extended region, corroborating the m5C-RIP-sequencing results.

RNA modification reader proteins have been extensively proposed as pivotal effectors mediating downstream regulations on modified mRNAs 5,8. Given this, we further investigated if RNA m5C readers make contributions to the regulating process of PRKACA. RNA pull-down results indicated that YBX1 particularly binds to PRKACA 3'UTR region, while the other two readers ALYREF and RAD52 as well as control protein GAPDH have no interaction with it (Figure 7J-K). Deletion of the methylated region or knocking down Nsun2 unexceptionally attenuated the association between YBX1 and PRKACA 3'UTR (Figure 7J-K), corroborating that YBX1 binds to PRKACA mRNA as an m5C reader. In keeping with this, RNA immunoprecipitation with differed readers showed that YBX1 specifically enriched PRKACA mRNA and Nsun2 knockdown sharply impaired the enrichment (Figure 7L). Moreover, silencing of YBX1 significantly reduced PRKACA expression (Figure 7M) and even abolished its increase due to Nsun2 overexpression in HL-1 cardiomyocytes (Figure 7N). These findings suggest that the RNA m5C reader YBX1 plays a central role in mediating the regulatory events post-methylation of PRKACA mRNA by Nsun2. Overall, these mechanistic explorations proposed that PRKACA mRNA methylation by Nsun2 promotes PRKACA translation in a YBX1-dependent manner.

### Nsun2 deficiency hampers the pressure overload-induced activation of PKA signaling in cardiomyocytes

**Figure 8:**
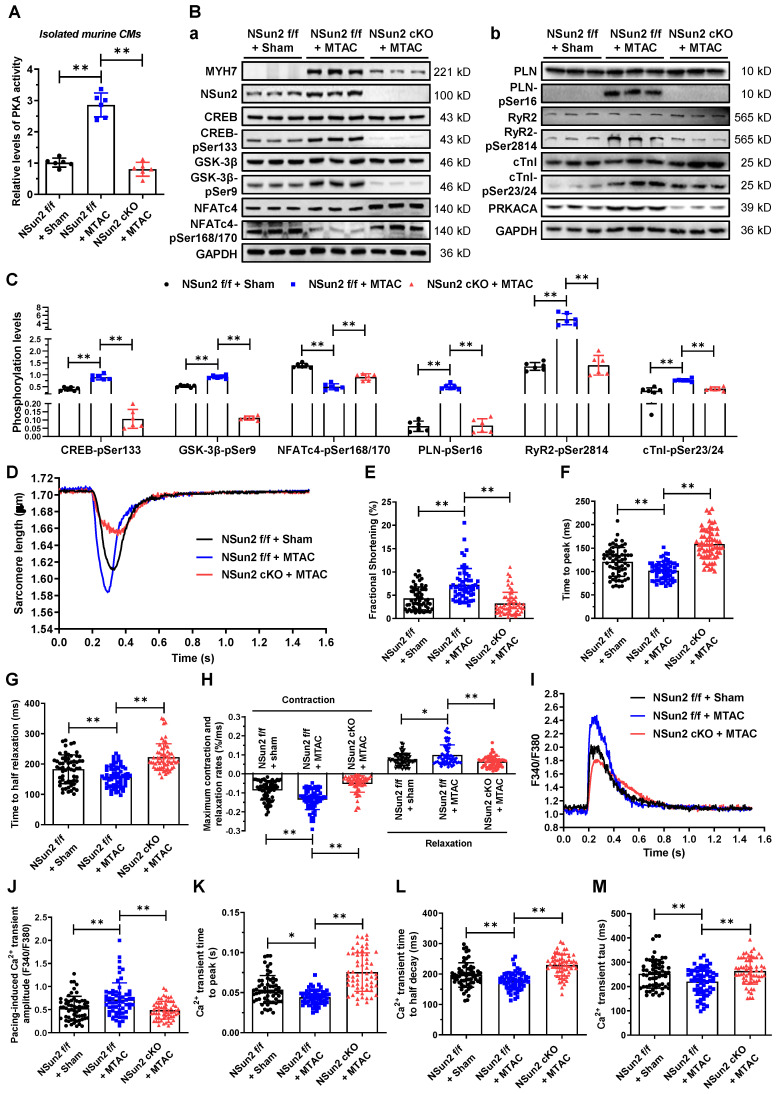
**Nsun2 deficiency inhibits cardiomyocyte hypertrophy-associated activation of PKA signaling.** Mice were divided into three groups including 'Nsun2 f/f + Sham', 'Nsun2 f/f + MTAC' and 'Nsun2 cKO + MTAC'. The following analysis was performed at three weeks after surgery. **(A)** Statistical analysis of relative PKA activity in isolated cardiomyocytes from the three indicated groups; n = 6 for each group. **(B)** Representative immunoblot of the expression and phosphorylation of PKA signaling pathway-dependent cardiac hypertrophy-related proteins (a) and calcium homeostasis-related proteins (b) in the cardiomyocytes of the three groups. **(C)** The densities of the signals in (B) were scanned and plotted; n = 6 for each group.** (D)** Representative traces of sarcomere length in isolated cardiomyocytes in the three indicated groups. **(E to H)** The percent of sarcomere FS (E), time to peak (F), time to half contraction (G), and maximal contraction/relaxation rate (H) were compared in isolated cardiomyocytes in the three indicated groups; n = 60 cells from 3 mice per group. **(I)** Representative Ca^2+^ transient tracing in the isolated cardiomyocytes in three indicated groups. **(J to M)** Pacing-induced Ca^2+^ transient amplitude (J), Ca^2+^ transient time to peak (K), Ca^2+^ transient time to half decay (L) and tau of Ca^2+^ transient decay (M) were compared among the three indicated groups; n = 60 cells from 3 mice per group. Data were analyzed by one-way ANOVA followed by Bonferroni's post hoc tests and presented as the means ± SEMs. *P < 0.05, **P < 0.01.

PKA signaling has been profoundly studied of versatile roles in heart hypertrophic remodeling 38. To illuminate the impacts of Nsun2 on PKA signal transmission in cardiomyocyte hypertrophy, a plethora of cell biological events downstream of PKA signaling were examined. First, we assessed the gross changes in PKA activity within immediately prepared murine cardiomyocytes and found that Nsun2 knockout sharply blunted the MTAC-induced increase in PKA activity (Figure 8A), emphasizing the importance of PRKACA expression regulation in cardiomyocyte hypertrophy-associated PKA activation. Subsequently, we examined changes in the phosphorylation levels of well-characterized PKA substrates in these myocytes. Immunoblotting showed that Nsun2 knockout abrogated MTAC-provoked elevations in the phosphorylation levels of CREB (Ser133) and GSK-3β (Ser9) as well as reductions in NFATc4 (Ser168/170) phosphorylation levels, suggestive a transcriptional inactivation for hypertrophy genes 38, as supported by the observed expression changes of MYH7 (Figure 8B-a and 8C). Similarly, MTAC-induced phosphorylation of other PKA effectors involved in calcium handling (PLN, Ser16; RyR2, Ser2814) and sarcomere motions (cTnI, Ser23/24) was repressed after Nsun2 depletion in cardiomyocytes (Figure 8B-b and 8C). We next tested the effects of Nsun2 deficiency on myocyte contractility upon pressure overload in freshly isolated ventricular myocytes. MTAC-induced inotropic and lusitropic effects in cardiomyocytes were both impaired in the absence of Nsun2, as supported by weakened sarcomere responses (Figure 8D), decreased FS (Figure 8E), and increased contraction and relaxation times and magnitudes (Figure 8F-H). Correspondingly, MTAC caused significant increases in the amplitudes of Ca^2+^ transients, whereas MTAC had little effect on Ca^2+^ handling in Nsun2-deficient myocytes (Figure 8I-M). Taken together, these results reflected PKA signaling blockade in Nsun2-knockout cardiomyocytes, consistent with findings in cardiomyocytes constitutively expressing a PKA inhibitor peptide (PKI) 28.

### Nsun2-PRKACA regulation is required to initiate the cardiomyocyte hypertrophy program

**Figure 9:**
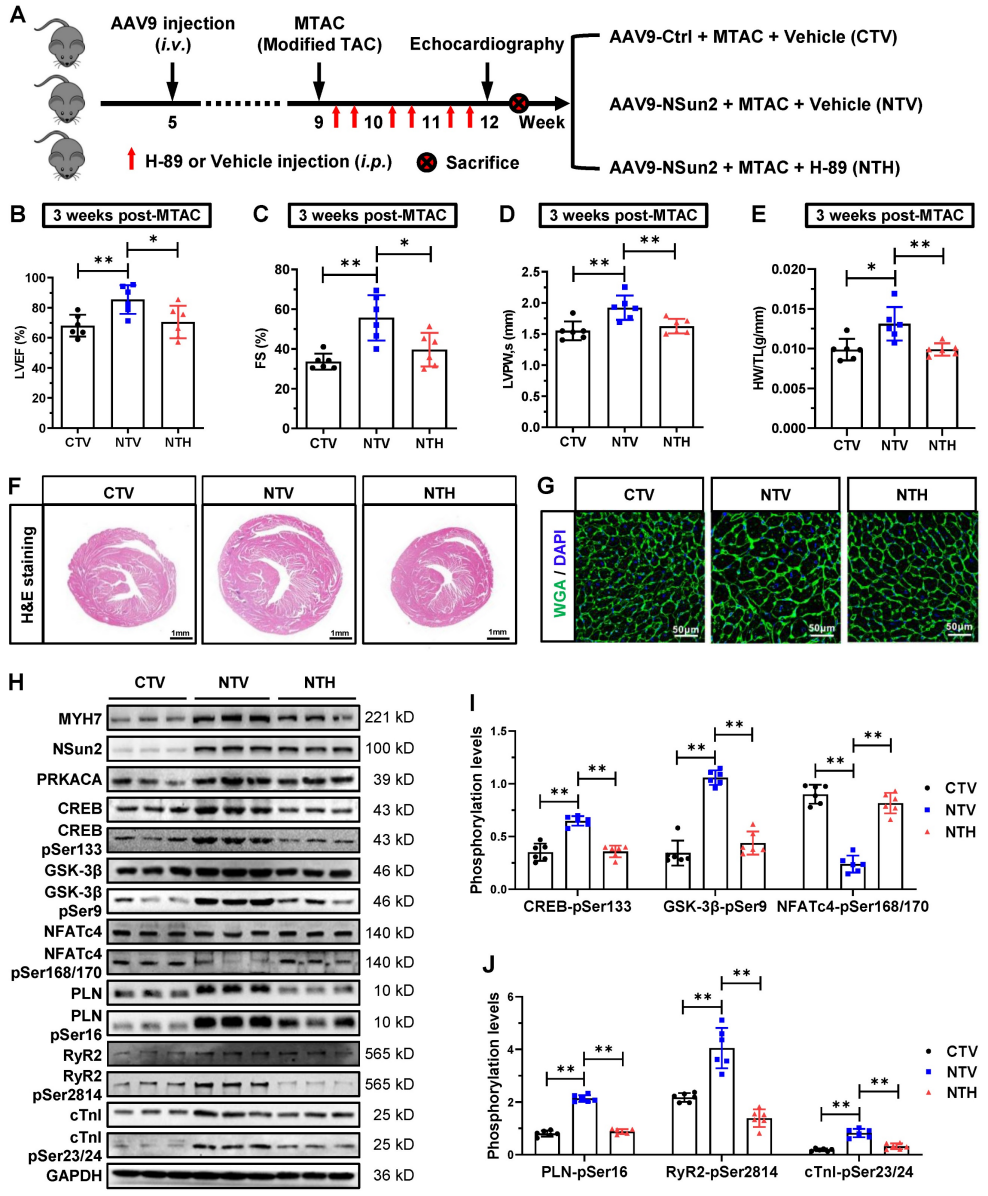
** Myocardium-specific overexpression of Nsun2 sensitizes the heart to a hypertrophic response via PKA signaling.** (A) Schematic diagram of experimental procedure. Briefly, 1×10^11^ vg in a total volume of 200 μL per animal was given at the 5th week after birth. MTAC was administered at the 9th week, and H89 (10 mg/kg twice a week, i.p.) was given at the indicated time (red arrow) until the 12th week.** (B to D)** Summarized echocardiographic measurements of left ventricular EF (B), FS (C), and LVPW,s (D) at the 12th week in the indicated groups; n = 6 for each group. **(E)** Statistical results of HW/TL values for the indicated groups; n = 6 for each group. **(F)** Representative images of H&E staining for cardiac morphology. Scale bar, 1 mm. **(G)** Representative histological images of heart sections stained with WGA from the indicated group. **(H)** Representative immunoblot of the expression and phosphorylation of PKA signaling pathway-dependent cardiac hypertrophy-related proteins and calcium homeostasis-related proteins in the cardiomyocytes of the indicated groups. **(I and J)** The densities of the signals in (H) were scanned and plotted; n = 6 for each group. Data were analyzed by one-way ANOVA followed by Bonferroni's post hoc tests and presented as the means ± SEMs. *P < 0.05, **P < 0.01.

To elucidate the role of Nsun2-PRKACA regulation in cardiac pathological hypertrophy, two well-designed interference strategies were employed: direct inactivation of PKA and inhibition of Nsun2-mediated PRKACA mRNA methylation. Initially, Nsun2 was overexpressed specifically in murine myocardium via adeno-associated virus serotype 9 (AAV9) with an expression cassette under the control of the cTnT promoter. Following the protocol illustrated in Figure 9A, the mouse models were categorized into three groups: CTV (AAV9-Ctrl + MTAC + Vehicle), NTV (AAV9-Nsun2 + MTAC + Vehicle), and NTH (AAV9-Nsun2 + MTAC + H-89). After confirming the AAV9 infection efficiency (Figure S6A), a series of assessments for hypertrophic remodeling were conducted. The results indicated that myocardial overexpression of Nsun2 accelerated cardiac hypertrophic progression at 3 weeks postoperatively, whereas administration of the PRKACA inhibitor H-89 42,43 significantly impeded early compensatory hypertrophic alterations in heart function, ventricular thickness, chamber dimensions, HW/TL values, and morphology (Figure 9B-F). Concomitantly, cardiac myocyte enlargement, aggravated by Nsun2 introduction, was notably attenuated following H-89 administration (Figure 9G and Figure S6B). These findings underscore the indispensable role of PKA in mediating Nsun2 modulation in the cardiac hypertrophic response. Furthermore, ventricular myocytes were isolated to interpret the corresponding variations at molecular and cellular levels. Nsun2 overexpression markedly promoted the protein expression of PRKACA and MYH7 and protein phosphorylation of the aforementioned PKA substrates compared to those in CTV myocytes. However, apart from the enhancement of PRKACA expression, H-89 treatment significantly attenuated the molecular changes resulting from Nsun2 overexpression (Figure 9H-J). Unambiguously, these findings verified the substantial contribution of PKA signaling to Nsun2-mediated regulation of pressure overload-induced hypertrophic heart remodeling.

**Figure 10:**
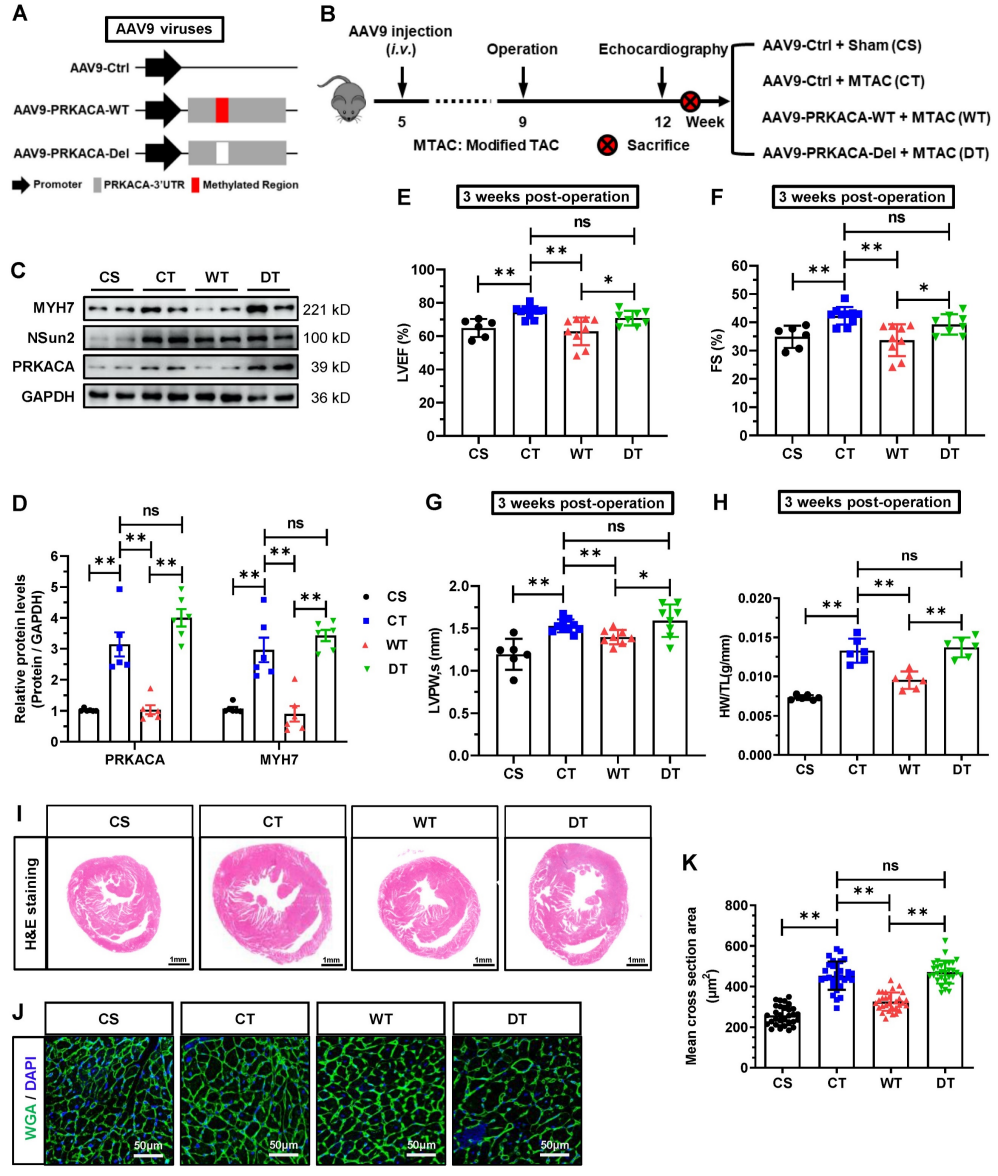
**Abrogation of the m5C methylation in endogenous PRKACA hindered the MTAC-induced cardiac hypertrophy. (A)** Schematic diagram of AAV9 virus constructs containing the 3' untranslated region (3'UTR) of PRKACA mRNA with (AAV9-PRKACA-WT) or without (AAV9-PRKACA-Del) Nsun2 methylation region. **(B)** Schematic diagram of experimental procedure. Briefly, 1×10^11^ vg in a total volume of 200 μL per animal was given at the 5th week after birth. MTAC was administered at the 9th week, and echocardiography was carried out at the 12th week. **(C)** Representative immunoblot of the expression of MYH7, Nsun2 and PRKAC in the cardiomyocytes of the four groups described in (B). **(D)** The densities of the signals in (C) were scanned and plotted; n = 6 for each group. **(E-G)** Summarized echocardiographic measurements of left ventricular EF (E), FS (F), and LVPW,s (G) at the 3 weeks post-operation in the indicated groups; n = 6 for each group. **(H)** Statistical results of HW/TL values for the indicated groups; n = 6 for each group. **(I)** Representative images of H&E staining for cardiac morphology. Scale bar, 1 mm. **(J and K)** WGA staining of ventricle sections from the indicated groups. WGA, green; DAPI, blue; Scale bar, 50µm (J) and the statistical results of mean cross-sectional areas of the stained cardiomyocytes; n ≥ 30 cells per group. Data were analyzed by one-way ANOVA followed by Bonferroni's post hoc tests and represented as means ± SEMs. ns, no significance; *P < 0.05, **P < 0.01.

To suppress the methylation of PRKACA by Nsun2 in cardiomyocytes, we exogenously introduced the 3'UTR RNA fragment of PRKACA (PRKACA-WT), which was designed to competitively inhibit the methylation of endogenous 3'UTR in PRKACA mRNA, into the murine myocardium utilizing the aforementioned AAV9 virus expression system. As illustrated in Figure 10A, the 3'UTR RNA lacking Nsun2 methylation regions (PRKACA-Del) was constructed as a control. Following AAV9 injection and surgical procedures, mouse models were categorized into four groups according to the experimental design (Figure 10B), comprising CS (AAV9-Ctrl + Sham), CT (AAV9-Ctrl + MTAC), WT (AAV9-PRKACA-WT + MTAC), and DT (AAV9-PRKACA-Del + MTAC). Immunofluorescence imaging identified a high and comparable AAV9 infection efficiency in murine hearts among the groups (Figure S7A). Subsequently, the overexpression of exogenous 3'UTR RNA fragments and the suppression of PRKACA mRNA methylation were primarily determined in the myocardium. Quantitative PCR results demonstrated that the 3'UTR RNA fragments bearing Nsun2 methylation regions or not were successfully overexpressed in WT and DT mouse hearts, respectively (Figure S7B). RNA m5C immunoprecipitation analysis using Langendorff-isolated cardiomyocytes indicated that the wild-type 3'UTR RNA fragment significantly reduced the methylation of PRKACA mRNA, whereas the 3'UTR RNA fragment deleting Nsun2 methylation regions had no impact on it (Figure S7C). Given the identified interference efficacies, we subsequently assessed the hypertrophic progression of these mouse models among the groups. First, PRKACA-WT fragment decreased MTAC-induced cardiomyocyte expression of both PRKACA and MYH7, but did not affect the induction of Nsun2 expression (Figure 10C-D). Corresponding to the molecular changes, we observed that the PRKACA-WT fragment significantly attenuated the pressure overload-triggered cardiac hypertrophic response, as evidenced by the alterations in the indices of LVEF (Figure 10E), FS (Figure 10F), LVPW (Figure 10G), and HW/TL (Figure 10H), as well as in the ventricle and myocardial cell size and morphology (Figure 10I-K). In contrast, the PRKACA-Del fragment failed to achieve this effect at the molecular, cellular, and morphological levels (Figure 10C-K). Based on these observations, Nsun2-PRKACA regulation was suggested to be indispensable for initiating the hypertrophic program in the heart.

## Discussion

Numerous studies have demonstrated that post-transcriptional regulation of RNA plays a substantial role in various heart diseases 44,45. Recent evidence suggests that RNA modifications are involved in the modulation of cardiac performance and function 6,7, adding another layer to our understanding of post-transcriptional regulation in the context of cardiac homeostasis. The dynamic modification of N6-methyladenosine (m6A) deposited on mRNAs has been proposed as a critical participant in the regulation of distinct heart remodeling and disease processes and has gained immense attention 6,7. Nonetheless, there have been very few investigations into other RNA methylation modifications in the heart that have also been well characterized in mRNAs with high abundance, such as m5C, m1A, m6Am, and m7G 4,5. In this study, we identified that the RNA cytosine methyltransferase Nsun2 is a critical RNA-modifying protein that maintains cardiomyocyte hypertrophy. Mechanistically, Nsun2 promotes the translation of the PKA catalytic subunit PRKACA via mRNA methylation, thus ensuring potent activation of PKA signaling.

Intriguingly, Nsun2, the most extensively studied m5C writer, exhibits functional similarities to the m6A writer METTL3 in regulating cardiac hypertrophic remodeling, as both are essential for maintaining a normal hypertrophic response in cardiomyocytes 46. Notably, cardiomyocyte-specific knockout of METTL3 does not affect the murine phenotype at baseline but begins to result in cardiac abnormalities at 8 months of age, consistent with progression towards heart failure 46. In contrast, cardiac depletion of Nsun2 had no influence on mouse phenotype at 8 months of age. Aging can induce heart hypertrophy as a modest and long-term chronic stressor, and different regulators that address cardiac aging pressure demonstrate differential responsiveness 47; the same is likely true for METTL3 and Nsun2. Several m5C methyltransferases have been identified to possess the capacity to modify, even identical, mRNA substrates 48,49. Therefore, the possibility cannot be excluded that in vivo, other RNA m5C-modifying enzymes may perform a compensatory role in Nsun2-deficient hearts in response to mild stress produced at the early stage of aging. As anticipated, phenotypes involving cardiac dysfunction in Nsun2-knockout mice were observed at an advanced age, when the heart was subjected to a more severe aging-induced burden. M5C is a dynamically regulated modification, similar to m6A, that can be erased by TET and ALKBH1 proteins and thus be reversibly regulated 50. Consequently, further investigation is required to determine whether changes in mRNA cytosine methylation levels during cardiac hypertrophy concurrently involve m5C writers and erasers.

PKA activation depends primarily on the free levels of PKA-C released from either the PKA holoenzyme or other PKA-C-trapping components such as the I-κB-PKA-C complex 15,16. In the past few decades, considerable attention has been paid to the modulation of the activating modes by which PKA-C dissociates from complexes. However, limited knowledge exists regarding the regulation of PKA-C gene expression, which directly dictates the free concentration of PKA-C, particularly at the post-transcriptional level. Our investigation revealed that the RNA epigenetic modification m5C installed by Nsun2 significantly enhances PRKACA translation, through which PKA activation can be potently achieved in cardiomyocytes exposed to hypertrophic stresses. These findings suggest that epitranscriptomic deposition of m5C marks on PRKACA mRNA to regulate PRKACA expression levels may contribute significantly to the activation of PKA signaling in cardiac hypertrophic remodeling.

Our research revealed that Nsun2 deficiency significantly reduced PKA activity and subsequent signaling cascades, resulting in distinct alterations in myocyte geometry and contractility, reminiscent of the phenotypes observed in cardiomyocytes constitutively expressing PKI 28. Similarly, cardiac PKA ablation by PKI does not induce functional or morphological abnormalities in mice at baseline, even at 12 months of age, but impairs myocardial cell basal contractility and cardiac adaptation to pathological stress 28, which corroborates our observations in Nsun2-knockout mice. In contrast, myocardial overexpression of Nsun2 significantly accelerated pressure overload-triggered cardiac hypertrophy via potentiated PKA signaling, consistent with the development of hypertrophy in the myocardium with constitutive overexpression of PRKACA 51. Such momentous discoveries unveiled by the straightforward manipulation of cardiac PRKACA in previous studies provide strong support for the conclusion that Nsun2 maintains the cardiac hypertrophic program primarily through PKA signaling. Cardiac myocyte hypertrophy is considered an important signal for the development of cardiac maladaptive remodeling and heart failure 1. Although knocking out Nsun2 greatly reduces myocardial hypertrophy, it exacerbates heart dysfunction rather than ameliorating or preserving heart function. In light of these findings, we postulated that there are other unidentified Nsun2 targets that mediate the effects of Nsun2 on cardiac homeostasis. These potential targets of functional or expression impairment due to Nsun2 deficiency, along with insufficient activation of PKA signaling, contribute to the development of cardiac function decompensation in Nsun2 knockout mice under stress. With the identification of additional Nsun2 substrates in the myocardium, the understanding of how Nsun2 regulates cardiac function and homeostasis should be further elucidated.

In summary, our investigation identified the RNA cytosine methyltransferase Nsun2 as a crucial regulator of cardiac functional homeostasis in response to hypertrophic stimuli. Elevated expression of Nsun2 in the hypertrophied myocardium facilitates PRKACA translation via mRNA cytosine methylation, thereby maintaining sufficient PKA activation during cardiac hypertrophic remodeling. Myocardial depletion of Nsun2 compromises PKA signaling cascades and exacerbates heart failure progression under conditions of sustained pressure overload (Graphical abstract). Inhibition of PKA-C mRNA methylation or its activity significantly attenuated cardiac hypertrophic responses, underscoring the central role of Nsun2-PRKACA regulation in maintaining normal cardiac function.

## Materials and methods

Detailed materials and methods are provided in the Supplementary materials.

### Ethics statement

Procurement of human heart tissues strictly conformed to the principles outlined in the Declaration of Helsinki and was approved by local Research Ethics Committee of the First Affiliated Hospital of Zhejiang University (2022-Y-1159). Written informed consent was obtained from all participants. All animal studies were performed in accordance with the Guideline for Animal Care and approved by the Animal Care and Utilization Committee of Central China Fuwai Hospital of Zhengzhou University (ZZU-LAC20180518[09]) and complied with the National Institutes of Health Guide for the Care and Use of Laboratory Animals (NIH publication No 85-23). After terminal studies at the indicated time points, animals were sacrificed under 3% isoflurane inhalation followed by cervical dislocation.

### Statistical analysis

Data were presented as mean ± SEM or SDs unless otherwise specified. One-way analysis of variance (ANOVA) was used to determine statistical significance for experiments with more than two groups followed by Bonferroni's post hoc tests. Comparison between two groups was evaluated by an unpaired Student's *t*-test. P-values less than 0.05 were considered statistically significant and assigned in individual figures.

### Data availability

The data and study materials will be available upon reasonable request. Human data are unavailable to other researchers because of our institution's data-protection policy. The raw data used for analysis of RNA m5C-RIP sequencing can be found in GEO public repository (GSE234445).

## Supplementary Material

Supplementary figures and table legends.

Supplementary table 1.

Supplementary table 2.

Supplementary table 3.

## Figures and Tables

**Table 1:** Clinical information of human subjects enrolled in this study.

Parameter	Normal (n=5)	HCM patients (n=7)	Non-HCM hypertrophy patients (n=9)
General data			
Age (Years)	46.37±13.96	43.71±8.92	49.89±7.22
Male sex, n	40.00% (2)	42.86% (3)	44.44% (4)
BMI (kg/m^2^)	20.92±1.99	22.11±2.61	23.08±2.44
Tobacco use, n	1	2	2
Hypertension, n	1	2	1
Coronary heart disease, n	0	0	0
Diabetes, n	0	0	0
Stroke or TIA, n	0	0	0
Cancers, n	0	0	0
Left ventricle			
Septal thickness, mm	9.22±0.58	16.44±3.07	17.97±2.51
Maximum LVOT gradient, mmHg	16.14±4.06	75.92±12.82	66.43±12.42
LVPW, mm	9.86±1.06	14.84±1.32	14.59±1.48
Ejection fraction (%)	58.20±6.42	74.71±6.07	68.33±9.10
LVID,d, mm	46.40±3.05	35.14±3.72	36.43±3.85
Aortic valve area (cm²)	3.62±0.24	3.45±0.36	0.57±0.12
NYHA functional class			
Class I or II, n	0	5	5
Class III or IV, n	0	2	4
Serological data			
NT-proBNP, ng/L	190.80±78.52	845.56±518.38	1059.39±682.7
Creatinine, μmol/L	74.45±9.87	71.29±14.86	75.78±13.48
Hemoglobin, mg/dL	118.31±6.82	120.14±11.42	119.78±9.56
Drug therapy			
Beta-blocker, n	1	7	1
Calcium channel blocker, n	1	6	1
Loop diuretic, n	0	1	0

HCM, hypertrophic cardiomyopathy; Non HCM hypertrophy, aortic stenosis with left ventricular hypertrophy; BMI, body mass index; TIA, transient ischemic attacks; LVOT, left ventricular outflow tract; LVID,d, left ventricular end-diastolic diameter.
