# Supplementary material for: Nsun2 controls cardiac homeostasis and hypertrophic response by regulating PRKACA expression
Source: Theranostics. 2025 Jan 20;15(6):2393–412. doi: 10.7150/thno.104441 (PMC11840729; doi:10.7150/thno.104441)
Supplement: Supplementary file 1 — Supplementary figures and table legends. [file thnov15p2393s1.pdf]

Figure S1

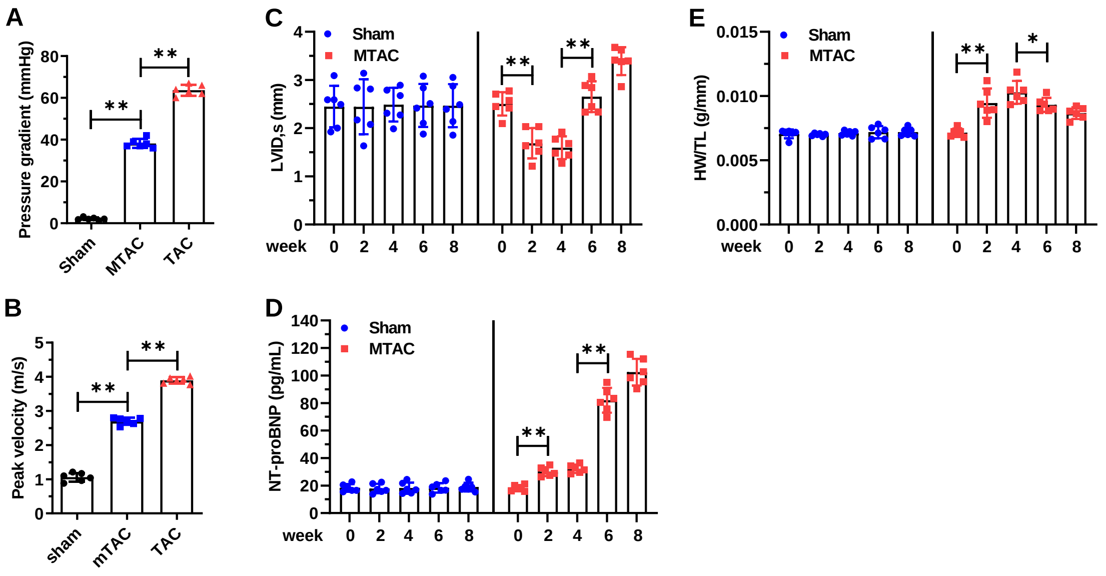

Figure S1 MTAC reduces the pressure gradient after aortic constriction surgery and prolongs the compensatory period of myocardial hypertrophy. (A and B) Peak pressure gradients (A) and peak velocity (B) distal to the constriction were calculated from Doppler velocities after 1 week from Sham, MTAC (constriction with the diameter of 0.51 mm) and traditional TAC (constriction with the diameter of 0.41 mm). n = 6 for each group. Data were represented as means ± SEMs and analyzed by unpaired Student's *t*-test. ***P* < 0.01. **(C to E)** Summarized echocardiographic measurements of left ventricular diameter at end-systole (LVID, s) (C), serological NT-proBNP levels (D), and heart weight/tibia length ratio (HW/TL) (E) from Sham and MTAC groups at the indicated weeks. n = 6 for each group. Data were analyzed by one-way ANOVA followed by Bonferroni's post hoc tests and represented as means ± SEMs. **P* < 0.05, ***P* < 0.01.

Figure S2

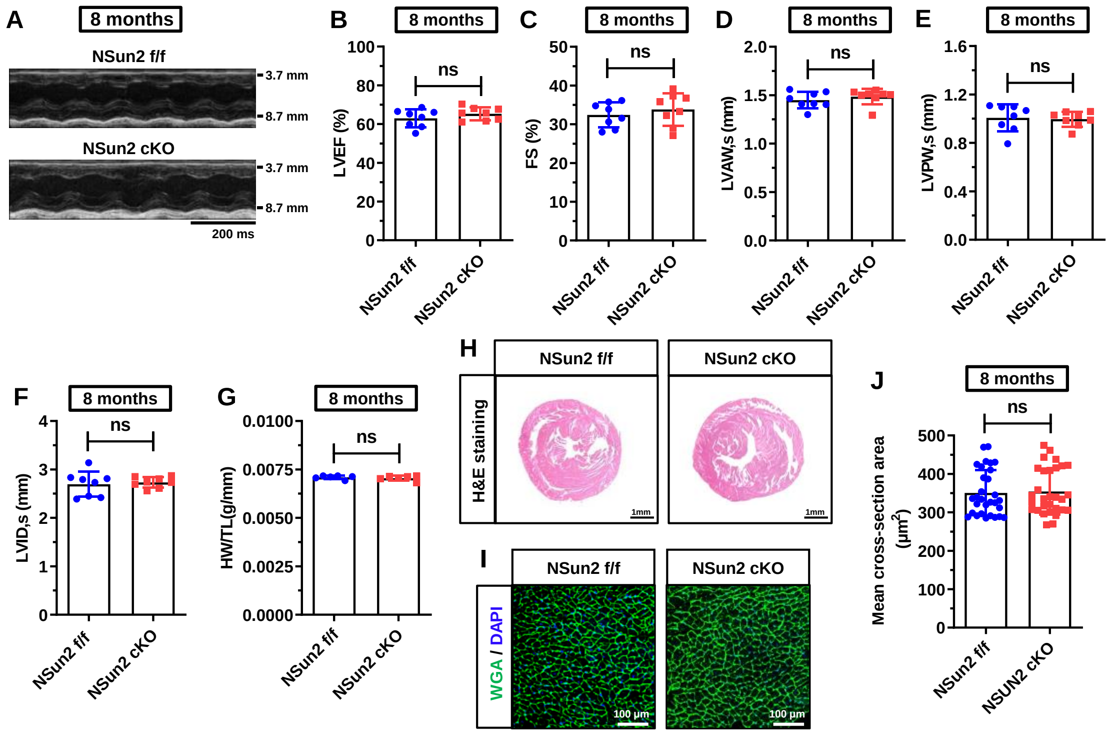

Figure S2 Myocardium-specific Nsun2 knockout does not affect cardiac function and cell morphology in basal state. (A) Representative echocardiographic images of control (Nsun2 f/f) and cardiomyocyte-specific Nsun2 knockout (Nun2 cKO) mice at 8 months of their age. **(B to F)** Summarized echocardiographic measurements of left ventricular EF (B), FS (C), LVAW,s (D), LVPW,s (E), and LVID,s (F) of the mice described in (A), n = 8 for each group. **(G)** Statistical results of murine HW/TL values, n = 8 for both groups. **(H)** Representative images for H&E staining of ventricles. Scale bar, 1 mm. **(I and J)** Representative pictures of WGA staining of murine heart sections (I) and quantification of the mean cross-sectional areas of the stained cardiomyocytes (J); n ≥ 30 cells per group. Data were analyzed by unpaired Student's *t*-test. ns, no significance.

Figure S3

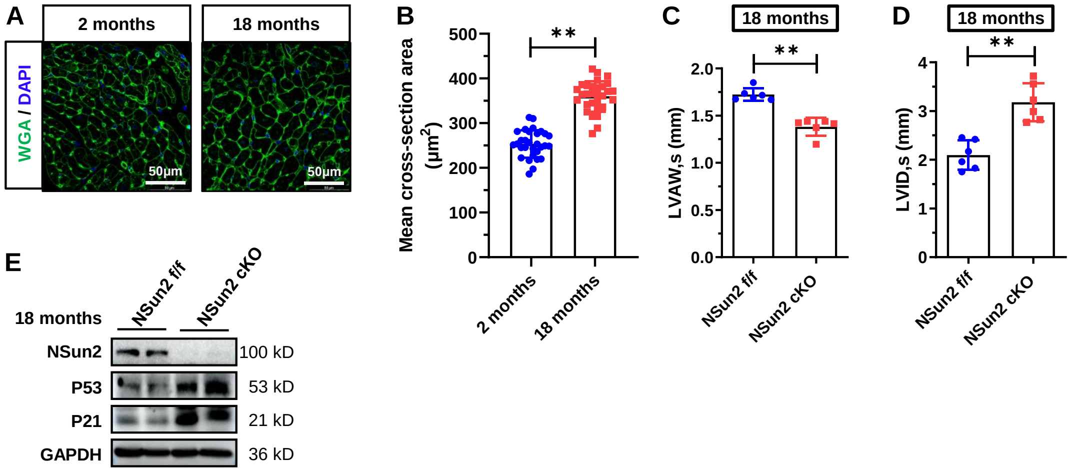

Figure S3 Nsun2 ablation eliminates aging-induced myocardial cell hypertrophy. (A and B) Representative pictures of WGA staining of Nsun2 f/f murine heart sections (A) and quantification of the mean cross-sectional areas of the stained cardiomyocytes (B) at 2 or 18 months; n ≥ 30 cells per group. Data were analyzed by unpaired Student's *t*-test. $^{**}P < 0.01$. **(C and D)** Echocardiographic measurements of LVAW,s and LVID,s in Nsun2 f/f and Nsun2 cKO mice at 18 months of their age. n = 6 for each group. Data were analyzed by unpaired Student's *t*-test. $^{**}P < 0.01$. **(E)** Immunoblotting analysis of the expression changes of Nsun2, P53, P21 and GAPDH in heart tissues of the 18-month old mice.

Figure S4

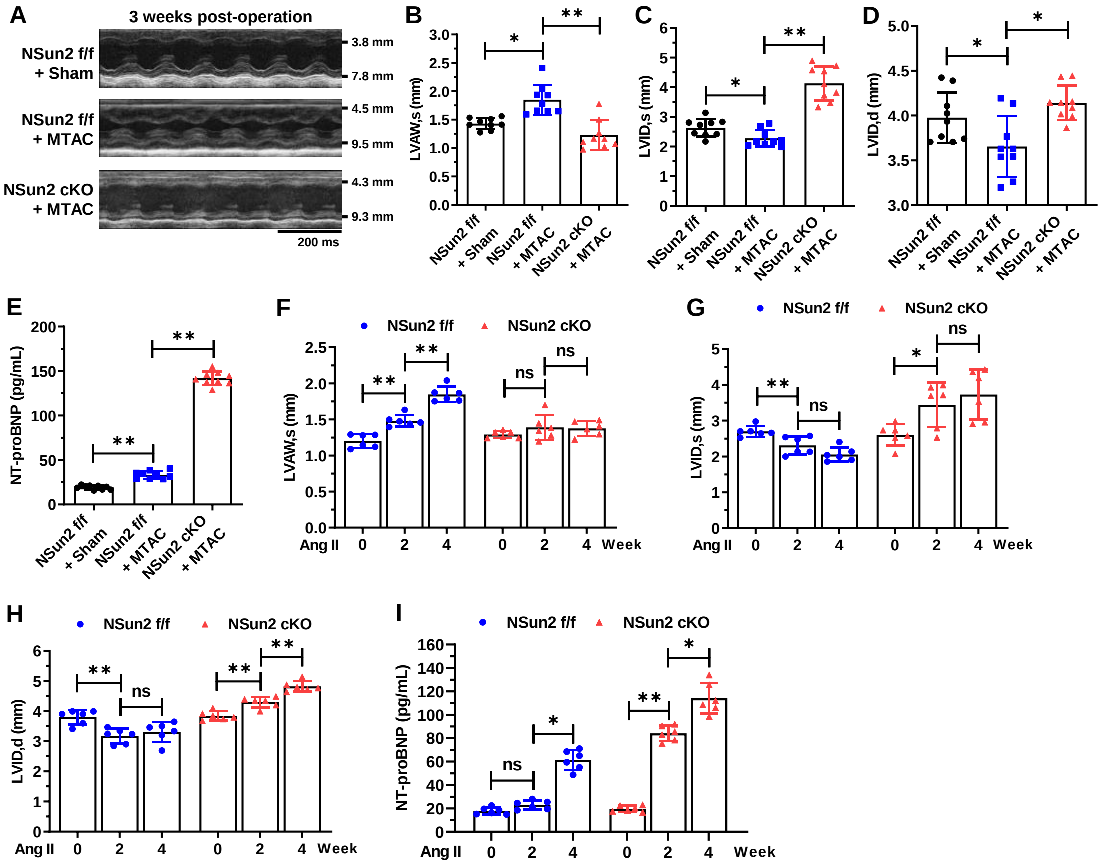

Figure S4 Myocardium-specific Nsun2 ablation interrupts the heart hypertrophic response and predisposes the heart to failure. (A) Representative echocardiographic images of mice from the three groups. **(B to E)** Summarized echocardiographic measurements of LVAW,s (B), LVID,s (C), and LVID,d (D) and serological NT-proBNP levels (E) of the mice described in (A), n = 9 for each group. **(F to I)** Summarized echocardiographic measurements of LVAW,s (F), LVID,s (G) and LVID,d (H) and serological NT-proBNP levels (I) of Nsun2 f/f and Nsun2 cKO mice administrated by subcutaneous infusion of AngII at a dose of 1000 ng/kg/min for 4 weeks, n = 6 for each group. Data were analyzed by one-way ANOVA followed by Bonferroni's post hoc tests and represented as means ± SEMs. *P < 0.05, **P < 0.01.

Figure S5

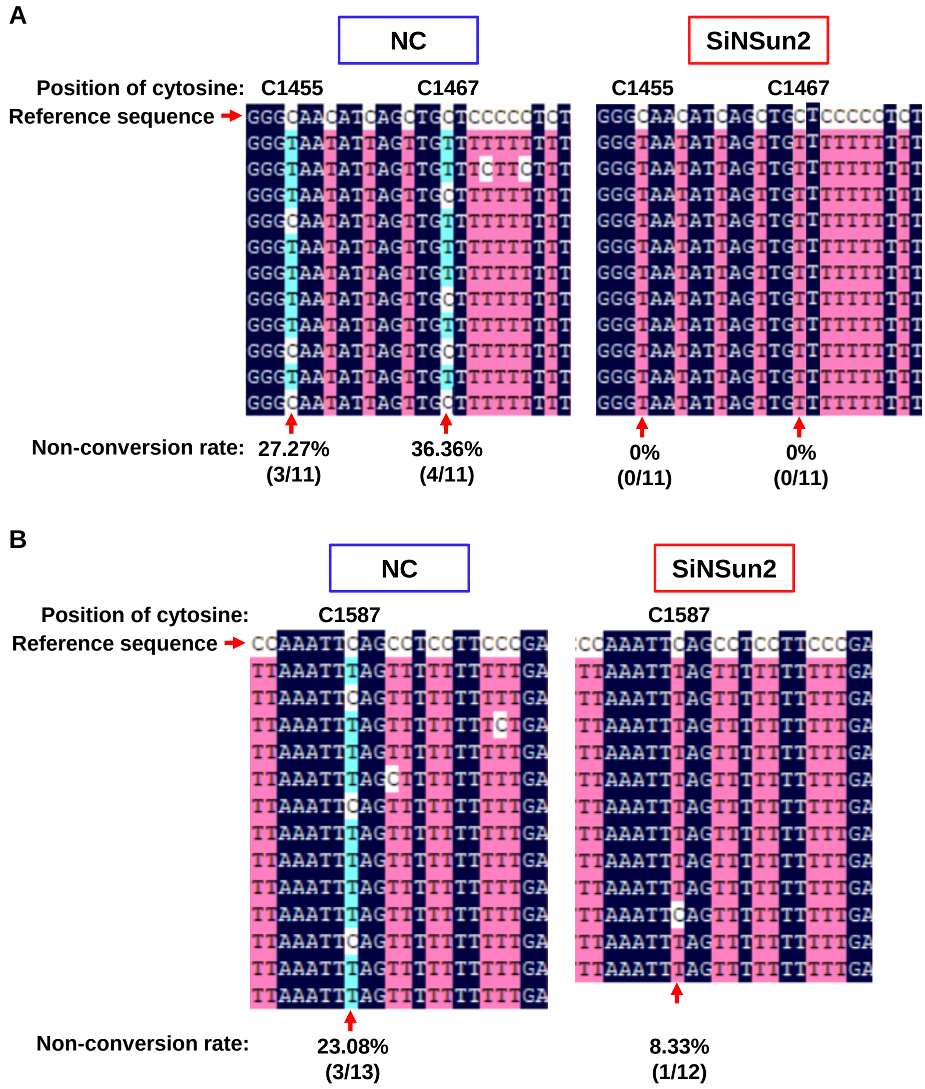

Figure S5 Identification of cytosine methylation (m5C) sites harbored within the 3'untranslated region (3'UTR) of PRKACA mRNA. (A and B) RNA bisulfite PCR (BS-PCR) assays were conducted utilizing RNA extracted from HL-1 myocardial cells subjected to treatment with or without Nsun2 siRNA.

Figure S6

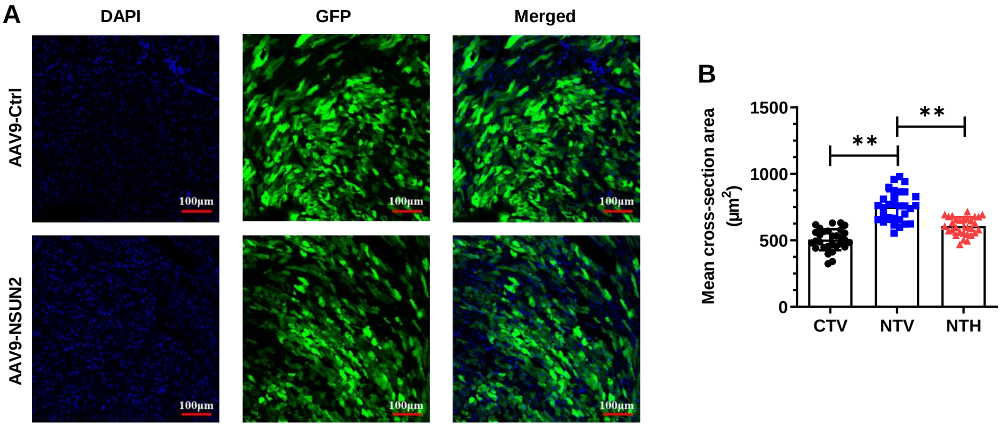

Figure S6 Myocardium-specific overexpression of Nsun2 sensitizes the heart to a hypertrophic response via PKA signaling. (A) Representative immunofluorescence images of heart tissue sections from mice injected with AAV9-ctrl- or AAV9-Nsun2-expressing cassette. **(B)** Statistical analysis of the mean cross-sectional areas of the stained cardiomyocytes from Figure 9A; $n \geq 30$ cells per group. Data were analyzed by one-way ANOVA followed by Bonferroni's post hoc tests and represented as means ± SEMs. $^{**}P < 0.01$.

Figure S7

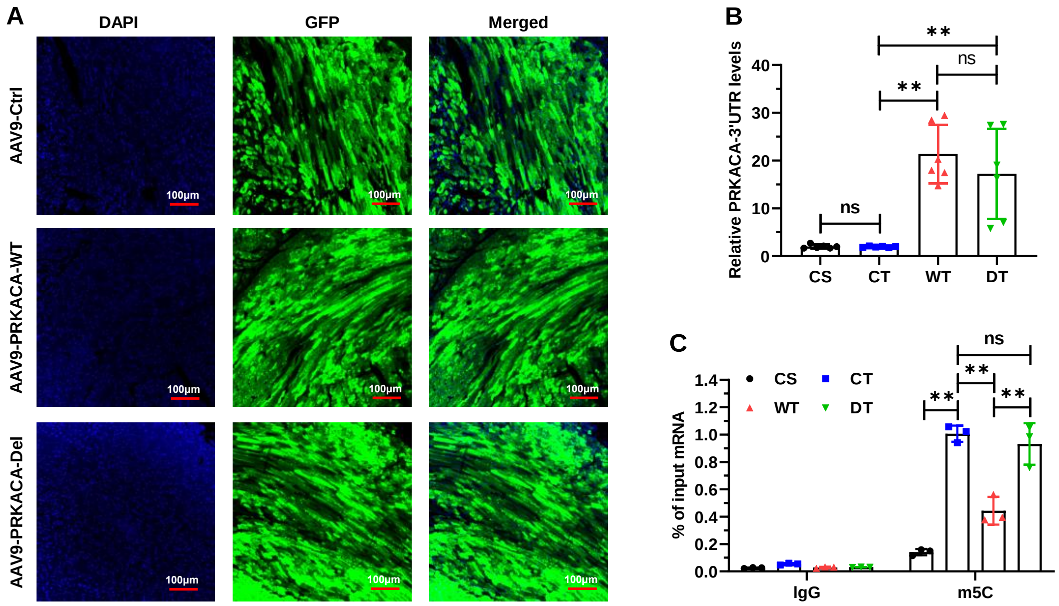

Figure S7 Abrogation of the m5C methylation in endogenous PRKACA hindered the MTAC-induced cardiac hypertrophy. (A) Representative immunofluorescence images of heart tissue sections from the indicated mice revealed the viral transfection efficacy. **(B)** Quantitative RT-PCR analysis was performed to assess the expression levels of PRKACA 3'UTR fragment. n=6 for each group. **(C)** RNA extracted from the indicated murine cardiomyocytes was subjected to m5C-RIP-qPCR to evaluate the methylation level of PRKACA. Data were analyzed by one-way ANOVA followed by Bonferroni's post hoc tests and represented as means ± SEMs. ns, no significance. ***P* < 0.01.

Detailed Methods and Materials

Human myocardium samples:

Hypertrophic heart tissue samples were obtained from patients with hypertrophic cardiomyopathy (HCM) or aortic stenosis with left ventricular hypertrophy (Non-HCM), both combined with severe left ventricular outflow tract obstruction (outflow tract pressure gradient ≥ 50 mmHg) and underwent modified Morrow surgery for partial ventricular muscle resection. Tissue of healthy adult human hearts was obtained from donor hearts that were not utilized for heart transplants. Overview on human heart samples used in this study is provided in Table 1. All research protocols were approved by the local ethics board of the First Affiliated Hospital of Zhejiang University (2022-Y-1159) in accordance with the principles outlined in the Declaration of Helsinki. Written informed consent was obtained from all participants. The myocardium samples were cryopreserved in liquid nitrogen until RNA and protein samples were extracted.

Reagents and antibodies:

AngII (#HY-13948), PE (#HY-B0471) and H89 (#HY-15979A) were purchased from MCE. MYH7 (#sc-53090), Phospho-NFATc4 (Ser168/170) (#sc-135771) and RAD52 (sc-365341) antibodies were purchased from Santa Cruz Biotechnology (Santa Cruz, CA, USA). Nsun2 (#36103S), Phospho-GSK-3β (Ser9) (#5558S), GSK-3β (#12456S), CREB (#9197S), Phospho-CREB (Ser133) (#9198S), Troponin I (#4002S), Phospho-Troponin I (Cardiac) (Ser23/24) (#4004S), NFATc4 (#2183S) and PKA C-α (PRKACA) (#4782S) antibodies were purchased from Cell Signaling Technology (Beverly, MA, USA). RYR2 (#A0298), PLN (A23750), and GAPDH antibodies (#AC001) were purchased from Abclonal (Wuhan, Hubei, China). Phospho-RyR2 (Ser2808) (#PA5-105712) and RyR2 (#PA5-87416) antibodies were purchased from ThermoFisher. YBX1 (ab76149), ALYREF (ab202894), PLN (#ab219626) and Phospho-PLN (phospho S16) (#ab92697) were purchased from Abcam (Massachusetts, USA). All antibodies were used according to the dilution ratio recommended in the instructions.

Mice：

$Nsun2^{flox/flox}$ mice were generated on the C57/BL6 background utilizing CRISPR/Cas9-based targeting and homology-directed repair by Cyagen Biosciences (Suzhou, Jiangsu, China). Male wild type and $Myh6\text{-}Cre^{ERT2}$ mice were purchased from Gempharmatech Co., Ltd (Nanjing, Jiangsu, China). $Nsun2^{flox/flox}$/ $Myh6\text{-}Cre^{ERT2}$ (Nsun2 cKO) mice were obtained through mating and breeding between $Nsun2^{flox/flox}$ mice and $Myh6\text{-}Cre^{ERT2}$ mice. In founder lines, PCR amplification was performed to verify genotyping, and the primers used were listed in Table S3. For conditional knockout of Nsun2 gene in Nsun2 cKO mice, cre recombinase was activated in male mice with 75 mg/kg tamoxifen (T-5648, Sigma) intraperitoneal injections for 5 days at the age of 8 weeks. Subsequent procedure was conducted seven days after last injection.

AAV9-PRKACA-WT, AAV9-PRKACA-Del, AAV9-Nsun2 or AAV9-ctrl virus were produced by Hanbio Biotechnology Co. (Shanghai, China) and delivered intravenously via tail vein

injection at a total of $1x10^{11}$ VG in a total volume of 200 μL per animal. Four weeks post-AAV9 injection, cardiac tissue was collected for immunofluorescence and western blot analysis to confirm AAV infection efficiency, and additional experiments were conducted.

Cell culture and siRNA transfection:

HL-1 and AC16 cells were purchased from the National Collection of Authenticated Cell Cultures (Shanghai, China) and maintained at 37 ℃ under an atmosphere of 5% CO_2 in DMEM medium containing 10% fetal bovine serum (Gibco), 100 U/ml penicillin, and 100 U/ml streptomycin. Cells were transfected with 25nM of Nsun2 siRNA (siNsun2) or negative control siRNAs (siNC) using Lipofectamine® RNAiMAX™ transfection reagent (Invitrogen, 11668–019) according to the manufacturer's instructions. siRNA sequences were listed in Table S3. Cells were collected 48-72 hours after transfection and then performed subsequent analysis.

Modified transverse aortic constriction (MTAC):

The modified transverse aortic constriction mouse model was performed as described previously with appropriate modifications(*1*). Briefly, mice at 8–10 weeks of age (body weight: 22–25g) were used and anesthetized with ketamine/xylazine (55mg/kg/3.3mg/kg) via intraperitoneal injection. When the animal was deeply anaesthetized, the skin anterior to the trachea was incised and muscle tissue was passively separated. Slightly lift and cut the sternum to the second rib, and pull to both sides to fully expose the aortic arch. The 6-0 nylon suture was used to tighten and ligation the 25-gauge needle (with an outer diameter of 0.51 mm) to complete transverse aortic constriction between the right innominate and left common carotid arteries of the aorta. The sham group underwent the same procedure, but without the application of a ligature. Suture the sternum and skin. The mice were transferred onto a heating pad and subjected to close monitoring. When the mice regained consciousness, they were administered buprenorphine (1 mg/kg subcutaneously) twice a day for 2 days. Pulsed wave Doppler-based echocardiography techniques were used 1 week after sham or MTAC surgery to determine the peak pressure gradient and peak velocity across the aortic constriction. Mice with a pressure gradient between 35-45 mmHg were used for further examination. All operators and analyzers were performed with randomization in a blinded manner to avoid mouse genotype preference.

AngII-induced cardiac hypertrophy:

Wild-type (WT), Nsun2 f/f, and Nsun2 cKO mice at 8–10 weeks of age were used to establish the cardiac hypertrophy model by subcutaneous infusion of AngII at a dose of 1000 ng/kg/min with Alzet 2004 pumps as described previously(*2*).

Echocardiography:

Echocardiograms were obtained using a Vevo 3100 Ultrasound System (VisualSonics, Toronto, Canada) equipped with a high-frequency (30 MHz) linear array transducer. Mice were

anaesthetized with isoflurane (3% for induction and 1–1.5% for maintenance) mixed in 1L/min O_2 via a facemask to maintain heart rates within the range of 400–600 beats/minute. The systolic and diastolic ventricular diameter and wall thickness were determined through the measurement of the two-dimensional M-mode obtained from a parasternal short-axis view at the papillary muscle level. All analyses were performed in a blinded manner with regard to mouse genotype.

Neonatal rat ventricular myocyte (NRVM) isolation and induction of cardiac hypertrophy:
The isolation and culture of neonatal rat ventricular myocyte (NRVMs) are described previously(*3*). Briefly, NRVMs were isolated from 1-3-day-old Sprague-Dawley rats. Hearts were extracted and rinsed with ice-cold PBS to eliminate any residual blood. Subsequently, the ventricles were dissected and minced into small fragments, which were then enzymatically dissociated in a digestion buffer consisting of 1mg/ml collagenase II and 0.125% trypsin. Shaking gently in a 37℃ water bath until the ventricular mass was completely disappeared. Digestion was terminated by adding a F12 medium containing 10%FBS. The supernatant was subjected to filtration through 100 mesh filters and centrifugation at 500rpm for 5 minutes. Discard the supernatant and re-suspended the cell pellet in complete medium (F12 medium (Gibco) with 10% fetal bovine serum (FBS) (Gibco), 20 U/mL penicillin, 20 μg/mL streptomycin) and plated in 100 mm tissue culture dishes. After incubation at 37℃ for 90 min, the cardiomyocytes still in suspension in the supernatant were collected, and the adherent fibroblasts were discarded. After centrifugation at 500rpm for 5min, cardiomyocytes were resuspended with complete medium containing bromodeoxyuridine (100 μmol/L, sigma) and placed in 35 mm tissue culture dishes at a density of $1x10^6$cells/dish. After incubation for 48h, the culture medium was substituted with serum-free medium for an additional 24h. Subsequently, AngII (1 μM) or PE (100 μM) were added and cultured for 48h to induce cardiac hypertrophy.

Langendorff isolation of murine cardiomyocytes:
Isolation of adult mouse cardiomyocytes using langendorff perfusion apparatus was performed as described previously(*4*). Briefly, mouse was intraperitoneally injected with heparin at 1000U/kg for 30min before starting the procedure. Subsequently, mice were anesthetized with 5% isoflurane and sacrificed by cervical dislocation. Hearts with approximately 2-3 mm of aorta were taken and placed to the langendorff perfusion system. Perfusing the heart with calcium-free medium (135 mM NaCl, 4 mM KCl, 1 mM $MgCl_2$, 10 mM HEPES, 0.33 mM NaH_2PO4, 10 mM D-(+)-Glucose, 10 mM BDM, 5 mM taurine, adjust pH to 7.4) for approximately 5 min to remove any remaining blood and immediately digesting through perfusion with enzyme cardiomyocyte isolation buffer (calcium-free medium with 525 U/ml Type II Collagenase and 0.67g/L BSA) for 10-15 min at 37 ℃. Once the heart has achieved a soft, swollen, and pinkish state, detach the heart from the cannulated syringe needle, trim off extra tissue and vessels, and shredded left ventricle into small pieces in enzyme cardiomyocyte isolation buffer. Pipette up and down softly until all the cells were detached. Cell suspension was filtered through a 100 mesh cell strainer. The cardiomyocytes

of natural sedimentation were collected after resting for 10min for three times.

Cardiomyocyte intracellular Ca^{2+} concentration, Ca^{2+} transients and sarcomere contraction and relaxation analysis:

Cardiomyocytes were isolated from $Nsun2^{flox/flox}$, $Nsun2^{flox/flox}$ + TAC or Nsun2-cKO + TAC hearts using the langendorff perfusion methods as described above. Use within 8 hours after cell separation. Ca^{2+} Concentration and Ca^{2+} transients were measured using Fura-2 AM (Byotime, S1052, China), a fluorescent Ca2+ indicator, under an inverted microscope in a 35±2℃ heating chamber. Randomly select rod-shaped, stationary myocardial cells with clear striations that respond to stimuli (1 Hz, 15 V, 4 ms pulse duration) with MyoPacer EP stimulator (IonOptix, Westwood, MA, USA). Measure the fluorescence emitted at 510 nm after alternating excitation at 340 and 380nm using the dual excitation fluorescence photomultiplier system (IonOpix), and record the Ca^{2+} transient in a single cell. Record the average background fluorescence in 10 cell groups without loading Fura-2 AM, and calculate the 340 nm/380 nm fluorescence ratios after subtraction. For sarcomere contraction and relaxation analysis, a rectangular region consisting of 15-20 sarcomeres was defined, and changes in sarcomere length were recorded with a variable-rate CCD video camera (MyoCam-S, IonOptix) connected to an inverted microscope (Olympus IX71). The Fast Fourier Transform (FFT) algorithm was used to record changes in sarcomere length during electrically paced contractions. Data from 20–30 twitches per cell were averaged. Measurements were conducted in over 60 cardiomyocytes for each group from n=6 animals, and IonWizard 6.5 software (IonOptix) was utilized for data analysis.

UID m5C-MeRIP-seq:

MeRIP experiment and high through-put sequencing and data analysis were conducted by Seqhealth Technology Co., LTD (Wuhan, China). Total RNAs were extracted from langendorff isolated cardiomyocytes using TRIzol Reagent (Invitrogen, #15596026) according to the instructions. DNA digestion was carried out after RNA extraction by DNase I. RNA quality was determined by examining A260/A280 with NanodropTM OneCspectrophotometer (Thermo Fisher Scientific Inc). RNA Integrity (RQN) was confirmed by Qsep 100 (BIOptic Inc). 200 μg total RNAs were used for polyadenylated RNA enrichment by VAHTS mRNA Capture Beads (VAHTS, cat. NO. N401-01/02）. 20 mM $ZnCl_2$ was added to mRNA and incubated at 95°C for 5-10min until the RNA fragments were mainly distributed in 100-200 nt. Then 10% RNA fragments was saved as “Input” and the rest was used for m5C immunoprecipitation. The specific anti-m5C antibody (Abcam, ab10805) was applied for m5C immunoprecipitation. RNA samples of both input and IP were prepared using TRIZol Reagent (Invitrogen, #15596026). The stranded RNA sequencing library was constructed by KC-Digital™ Stranded mRNA Library Prep Kit for Illumina® (#DR08502, Wuhan Seqhealth Co., Ltd. China) following the manufacturer’s instruction. The kit eliminates duplication bias in PCR and sequencing steps, by using unique molecular identifier (UMI) of 8 random bases to label the pre-amplified cDNA molecules. The

library products corresponding to 200-500 bps were enriched, quantified and finally sequenced on a MGISEQ-T7 instrument (MGI) in PE150 mode.

After reads were aligned onto the genome with Hisat2 as described previously(*5*), the unique comparison on the genome was finally obtained, and the comparison result of PCR duplicate was removed. And then two software programs, Piranha and ABLIRC, were used to perform peak calling. Piranha has been described elsewhere(*6*). The "ABLIRC" strategy for peak calling was conducted for each IP sample respectively. The process of peak calling is as follows: firstly, the whole genome was scanned with 5bp as a window and 5bp as a step from beginning of each chromosome. Peak is identified by requiring that the depth of the first window is 2.5 times for 8 consecutive windows on the genome, or medium depth is greater than 50. When the 8 consecutive windows is less than 4% of the max depth of this peak, the peak ends. At the same time, reads on each gene were randomly distributed to each gene for 500 times, and the frequency of peak depth of each peak was counted, so as to conduct significance analysis on identified peak and screen out the peak with significant peak (p-value < 0.05) or peak with maximum depth of a certain degree (≥ 10). Then, with the input samples as the control, the abundance difference analysis was conducted for the locations of these peaks, and the peak with IP abundance greater than 4 times (adjustable parameter) of input abundance was screened as the final combination peak. The target genes of IP were finally determined by the peaks and the binding motifs of IP protein were called by HOMER software (Heinz, Benner et al. 2010). Use bedtools to merge the peaks obtained for each sample, while retaining merge peaks that contain at least two samples. Calculate the number of reads per sample in the merge peak area, and then perform differential analysis using DESeq2. The peak with Fold change >1.5 and p value ≤ 0.01 is considered as the peak of difference between the two groups.

All sequencing raw data can be accessed at the Gene Expression Omnibus under accession GSE234445.

Nsun2-MeRIP:

Nsun2-MeRIP was performed as described in Harry George et al., 2017(*7*) with the following modifications: Specifically, HL-1 cells were transfected with mus-flag-Nsun2-mut plasmid to facilitate RNA substrate trapping. 48 hours later, the cell was harvested and treated with lysis buffer (50 mM Tris–HCl, pH 7.4, 100 mM NaCl, 1 % NP-40, 0.1 % SDS). The lysate was centrifuged at 20,000 × g for 20 min at 4 ℃ and the supernatant was used for the subsequent steps. Three micrograms of lgG or Flag antibody (Sigma, #F1804) was pre-bound to Protein A/G magnetic beads (ThermoFisher Scientific)) in lysis buffer at 4°C for 1 hour. The 300 micrograms of cell lysate was mixed with 500 μL lysis buffer and added to Protein A/G beads for 6 hours at 4°C. Samples were washed five times with high salt wash buffer (50 mM Tris–HCl, pH 7.4, 1 M NaCl, 1 mM EDTA, 1% NP-40, 0.1% SDS) and digested with Dnase I for 15 min at 37°C. Samples were then de-cross-linked at 55°C for 15min with proteinase K. RNA was extracted with Trizol Reagent (Invitrogen, #15596018CN). The reverse transcription of Input and IP-RNA was

conducted utilizing SuperScript III Reverse Transcriptase (ThermoFisher Scientific, #18080044) with random hexamers and enrichment determined by quantitative PCR.

M5C-RIP-qPCR:

Total RNAs from isolated cardiac cells from Nsun2$^{flox/flox}$ and Nsun2 cKO mice, as well as CS, CT, WT and DT mice, were treated with DNase I (ThermoFisher Scientific) and fragmented to 100–200 nucleotides with RNA Fragmentation Reagent (ThermoFisher Scientific, AM8740) and the fragmented RNA purified by ethanol precipitation. Five micrograms of IgG or anti-m5C antibody (Synaptic Systems, 202008) was pre-bound to 50uL Protein A/G magnetic beads at 4 ℃ for 2 h. Fragmented RNA was mixed with MeRIP buffer (10 mM Tris-HCl, 150 mM NaCl, 0.1% NP-40, pH 7.4, 0.4U/μL RNase inhibitor) and incubated with protein A/G magnetic beads for 4 hours at 4 ℃.Samples were washed four times in IP wash buffer. The bound RNAs were recovered by proteinase K digestion, phenol chloroform extraction and ethanol precipitation. Input and m5C-enrichment RNA were subjected to reverse transcription using Superscript III with random hexamers and analyzed by quantitative PCR. The pull-down efficiency was calculated as "% of input".

RNA cytosine methylation analysis:

RNA cytosine methylation analysis was performed using RNA bisulfite-PCR technique following the instructions of the commercial RNA methylation kit (R5001, Zymo Research). Initially, 1 μg of RNA was dissolved in 20 μL of RNase-Free water and mixed with 130 μL of RNA Conversion Reagent. The mixtures were incubated at 70 ℃ for 5 min, 54 ℃ for 45 min and held at 4 ℃. Subsequently, the samples were collected with Zymo-Spin IC Column and washed with 95-100% ethanol buffer. Next, the RNA was desulphonated with RNA Desulphonation buffer, washed with RNA wash buffer and eluted with RNase-Free water. Finally, the bisulfite-converted RNA was reverse-transcribed using the RevertAid First Strand cDNA Synthesis Kit (Thermo) with the stem-loop primers GCGTCTCAACTGGTGTCGTGGAGTCGGCAATTCAGTTGAGACGCCCT RRATACACCA and GCGTCTCAACTGGTGTCGTGGAGTCGGCAATTCAGTTGAGACGCA AATCTCCCTTTCC. PCR amplification was conducted using the primer pairs AGYGYTYYAGATAYTGGGGAAG and TGGTGTCGTGGAGTCGGC for fragment 1, and GGGYAGGGTAYGGAAAGAGG and TGGTGTCGTGGAGTCGGC for fragment 2. The PCR products were cloned into T plasmid using the pEAST-T&B Zero Cloning kit (CTB50, TrasnGene). The plasmids from individual clones were sequenced and aligned with PRKACA sequence. The cytosines that remained uncovered were considered methylated.

Luciferase activity:

For dual luciferase reporter gene assays, PRKACA 3'UTR sequences containing Nsun2 methylation regions or not were inserted into the pGL3-promoter vector (Promega) to generate pGL3-PRKACA-WT or pGL3-PRKACA-Del construct. HL-1 cells were transfected with

negative control (NC) or Nsun2 (SiNsun2) siRNA with Lipofectamine3000 (ThermoFisher Scientific, #L3000001). 48h later, pGL3-PRKACA-WT or pGL3-PRKACA-Del was co-transfected with Renilla luciferase construct into these cells (NC or SiNsun2). Dual luciferase activities were then measured using the dual-luciferase reporter gene system (Promega, #E1960) in accordance with the manufacturer's protocol. The relative luciferase activity was normalized to the Renilla luciferase internal control.

Polysome analysis:

Polysome analysis was performed as described previously(*8*). In brief, AC16 cells were transfected with siNC or siNsun2 for 48h, and CHX was introduced to the culture medium at a final concentration of 100 μg/ml 15 minutes prior to cell collection to stabilize ribosomes. Cells were harvested (10cm dish per sample, approximately 90% confluence) and resuspend in 500μl lysis buffer (20 mM HEPES-KOH (pH 7.4), 5 mM $MgCl_2$, 100 mM KCl, 200 μg/ml Heparin, 1% Triton X-100, 2 mM DTT, 100 μg/ml cycloheximide (CHX), 20 U/ml Rnase inhibitor, and Protease Inhibitor Cocktail) by repeated pipetting. Incubate on ice for 15 min, with vortexing every 2-3 min. Centrifuged at 13000g for 15 min at 4 ℃ and the supernatant was transferred to new ice-cold 1.5ml Rnase-free EP tubes. An equivalent volume of the supernatant was loaded onto a sucrose gradient ranging from 10-45%, and subsequently centrifuged for 2.5h using a SW41Ti rotor (Beckman Kurt) at 4 ℃, 36000rpm. The gradient was fractionated into 15 fractions using a gradient station (Biocomp), and continuous UV curves were recorded simultaneously. RNA extraction was carried out using TRIzol LS reagent according to the instructions. Specifically, 700μl of TRIzol LS was added to 700μl of each fraction and mixed. Additionally, 500ng Fluc mRNA was added to each fraction simultaneously with the addition of an equal volume of isopropanol to the chloroform extracted supernatant. Then, the subsequent RNA extraction step was continued. RNA was purified from each fraction and subjected to RT-qPCR analysis.

RNA pull-down assay:

RNA pull-down was performed as described in our previous study(*9*). Constructs pGL3-PRKACA-WT and pGL3-PRKACA-Del were used as a template for PCR amplification of different DNA fragments of the 3'UTR of PRKACA mRNA, respectively. For biotin pull-down assays, PCR-amplified DNA fragments were used as templates to *in vitro* transcribe biotinylated RNAs by using T7 RNA polymerase in the presence of biotin-UTP. One microgram of purified biotinylated transcripts was incubated with 100 μg of cytoplasmic extracts for 30 min at room temperature. The complexes were isolated with paramagnetic streptavidin-conjugated Dynabeads (Invitrogen). After the beads were washed thoroughly, the pull-down material was analyzed by western blot.

PKA activity examination：

PKA Activity was detected using the PKA Colorimetric Activity Kit (EIAPKA; ThermoFisher

Scientific) by measuring the phosphorylation level of a specific PKA substrate in accordance with the manufacturer's instructions. A quantity of 30ug of protein samples from langendorff isolated cardiomyocytes was employed for each assay.

Histology and imaging:

H&E and Masson's Staining were performed as described in our previous study(*10*). Briefly, the heart was fixed with 4% paraformaldehyde at room temperature for 24 hours for paraffin section or 2h for frozen section. Following dehydration and embedding with paraffin (for paraffin section) or OCT (for frozen section), sections were continuously sectioned with 5 μm along the short axis until the maximum short axis cross-section of the heart was reached. HE and Masson staining were performed to evaluate cardiac morphology and degree of fibrosis. After scanning using the digital scanning imaging system Olympus FV1000 (Olympus, Tokyo, Japan), quantitative analysis was performed from captured images by using Image J software (NIH, Bethesda, MD, USA).

WGA staining:

At the endpoint of the experiment, the hearts of different treatment groups of mice were harvested and embedded in paraffin and sectioned at a thickness of 5 μm. After conventional deparaffinization, fluorescein-conjugated wheat germ agglutinin (WGA; 5 μg/mL, 25530, AAT Bioquest, USA) dye solution was added and incubated at room temperature for 1h. Then, the sections were washed three times with PBS and mounted with an anti-fluorescence quenching medium containing DAPI. Subsequently, images were captured using the Olympus FV1000 digital scanning imaging system (Olympus, Tokyo, Japan). Image analysis and measurement of the myocardial cell lateral surface area were performed using Image J software (NIH, Bethesda, MD, USA).

Enzyme-linked immunosorbent assay (ELISA) analysis

At the endpoint of the experiment, blood samples of different treatment groups of mice were collected. Serum levels of N-terminal pro-Brain Natriuretic Peptide (NT-proBNP) were measured by the corresponding ELISA Kits (#E-EL-M0834, Elabscience Biotechnology Co., Ltd., Wuhan, China), according to the manufacturer's instruction.

Phalloidin staining:

Neonatal rat ventricular myocytes (NRVMs) were fixed with 4% paraformaldehyde at room temperature for 30min, followed by three times washes with PBS. Subsequently, the cells were permeabilized with PBS containing 0.5% Triton X-100 for 10 minutes. After washing with PBS for three times, the diluted CoraLite®594-Phalloidin (1:100, Cat No. PF00003, Proteintech, USA) solution was added and incubated at room temperature for 30min away from light. Cell nuclei were stained with DAPI and images were captured using the Olympus FV1000 digital scanning imaging system (Olympus, Tokyo, Japan).

Western blot:

Western Blot analysis was performed following standard procedure. Briefly, cells or tissues are lysised using RIPA buffer (containing phosphatase inhibitors and protease inhibitors). The 20-30ug protein was loaded and fractionated by SDS-polyacrylamide gel electrophoresis and transferred onto nitrocellulose membranes. Different antibodies are then used to detect the expression of respective target. After incubation with the HRP-conjugated secondary antibody, signals were detected by using chemiluminiscient detection system (iBright, USA). Intensities of the bands were analyzed and quantified with Image J software.

RNA isolation and polymerase chain reaction analysis:

RNA samples were extracted from tissues or cultured cells with TrizoL Reagent (Takara, #9108) and reverse transcribed to cDNA using SuperScipt Superscript II reverse transcriptase (ThermoFisher Scientific, #18090010) according to the manufacturer's instructions. Real time qPCR amplification reaction was performed using universal SYBR qPCR Master Mix (Vazyme, #Q511-02) and Bio-Rad CFX96TM Real-time SYSTEM. Primers used for RT-QPCR analysis were listed in Table S3.

References:

1. R. Tavakoli, S. Nemska, P. Jamshidi, M. Gassmann, N. Frossard, Technique of Minimally Invasive Transverse Aortic Constriction in Mice for Induction of Left Ventricular Hypertrophy. *Journal of visualized experiments : JoVE*, (2017).
2. L. Wang, Y. L. Zhang, Q. Y. Lin, Y. Liu, X. M. Guan, X. L. Ma, H. J. Cao, Y. Liu, J. Bai, Y. L. Xia, J. Du, H. H. Li, CXCL1-CXCR2 axis mediates angiotensin II-induced cardiac hypertrophy and remodelling through regulation of monocyte infiltration. *Eur Heart J* **39**, 1818-1831 (2018).
3. L. Hou, M. Deo, P. Furspan, S. V. Pandit, S. Mironov, D. S. Auerbach, Q. Gong, Z. Zhou, O. Berenfeld, J. Jalife, A major role for HERG in determining frequency of reentry in neonatal rat ventricular myocyte monolayer. *Circulation research* **107**, 1503-1511 (2010).
4. Y. Liu, D. E. Dostal, C. W. Tong, Isolation of Adult Mouse Cardiomyocytes Using Langendorff Perfusion Apparatus. *Methods in molecular biology* **2319**, 143-152 (2021).
5. D. Kim, J. M. Paggi, C. Park, C. Bennett, S. L. Salzberg, Graph-based genome alignment and genotyping with HISAT2 and HISAT-genotype. *Nature biotechnology* **37**, 907-915 (2019).
6. P. J. Uren, E. Bahrami-Samani, S. C. Burns, M. Qiao, F. V. Karginov, E. Hodges, G. J. Hannon, J. R. Sanford, L. O. Penalva, A. D. Smith, Site identification in high-throughput RNA-protein interaction data. *Bioinformatics* **28**, 3013-3020 (2012).
7. H. George, J. Ule, S. Hussain, Illustrating the Epitranscriptome at Nucleotide Resolution Using Methylation-iCLIP (miCLIP). *Methods in molecular biology* **1562**, 91-106 (2017).
8. E. S. Pringle, C. McCormick, Z. Cheng, Polysome Profiling Analysis of mRNA and Associated Proteins Engaged in Translation. *Current protocols in molecular biology* **125**, e79 (2019).
9. C. Liu, X. Cheng, J. Xing, J. Li, Z. Li, D. Jian, Y. Wang, S. Wang, R. Li, W. Zhang, D. Shao,

X. Ma, X. Chen, J. Shen, C. Shi, Z. Guo, W. Wang, T. Fan, L. Liu, H. Tang, CIRBP-OGFR axis safeguards against cardiomyocyte apoptosis and cardiotoxicity induced by chemotherapy. *International journal of biological sciences* **18**, 2882-2897 (2022).

10. X. Cheng, D. Jian, J. Xing, C. Liu, Y. Liu, C. Cui, Z. Li, S. Wang, R. Li, X. Ma, Y. Wang, X. Gu, Z. Ge, H. Tang, L. Liu, Circulating cardiac MicroRNAs safeguard against dilated cardiomyopathy. *Clinical and translational medicine* **13**, e1258 (2023).

Supplementary Table Legends

Table S1. The subset of protein-coding genes obtained from the comparative analysis between FF (Nsun2 f/f + Sham) and FT (Nsun2 f/f + MTAC) groups according to the screening criteria that the m5C peak enrichment degree was positively correlated with Nsun2 expression.

Table S2. The subset of protein-coding genes obtained from the comparative analysis between FT (Nsun2 f/f + MTAC) and CT (Nsun2 cKO + MTAC) groups according to the screening criteria that the m5C peak enrichment degree was positively correlated with Nsun2 expression.

Table S3. List of the primers and siRNAs involved in this study.
